# Determining Multi‐Component Phase Diagrams with Desired Characteristics Using Active Learning

**DOI:** 10.1002/advs.202003165

**Published:** 2020-11-23

**Authors:** Yuan Tian, Ruihao Yuan, Dezhen Xue, Yumei Zhou, Yunfan Wang, Xiangdong Ding, Jun Sun, Turab Lookman

**Affiliations:** ^1^ State Key Laboratory for Mechanical Behavior of Materials Xi'an Jiaotong University Xi'an 710049 China; ^2^ Los Alamos National Laboratory Los Alamos New Mexico 87545 USA

**Keywords:** Bayesian optimization, ferroelectrics, machine learning, materials informatics, multi‐component phase diagrams, shape memory alloys

## Abstract

Herein, we demonstrate how to predict and experimentally validate phase diagrams for multi‐component systems from a high‐dimensional virtual space of all possible phase diagrams involving several elements based on small existing experimental data. The experimental data for bulk phases for known systems represents a sampling from this space, and screening the space allows multi‐component phase diagrams with given design criteria to be built. This approach uses machine learning methods to predict phase diagrams and Bayesian experimental design to minimize experiments for refinement and validation, all within an active learning loop. The approach is proven by predicting and synthesizing the ferroelectric ceramic system (1‐*ω*)(Ba_0.61_Ca_0.28_Sr_0.11_TiO_3_)‐*ω*(BaTi_0.888_Zr_0.0616_Sn_0.0028_Hf_0.0476_O_3_) with a relatively high transition temperature and triple point, as well as the NiTi‐based pseudo‐binary phase diagram (1‐*ω*)(Ti_0.309_Ni_0.485_Hf_0.20_Zr_0.006_)‐*ω*(Ti_0.309_Ni_0.485_Hf_0.07_Zr_0.068_Nb_0.068_) designed for high transition temperature (*ω* ⩽ 1). Each phase diagram is validated and optimized through only three new experiments. The complexity of these compounds is beyond the reach of today's computational methods.

## Graphical abstract

A machine learning‐based approach is proposed to predict all possible phase diagrams of a given multi‐component system from a high‐dimensional virtual space. By quickly screening the space, a specific phase diagram with given design criteria can be constructed. Bayesian experimental design is then employed to refine the phase diagram with as few experiments as possible.

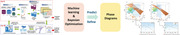

## 1 Introduction

Phase diagrams are fundamental to an understanding of the equilibrium structure, stability, and phase transitions of a metallic alloy or ceramic solid solution as a function of temperature and composition. They play a key role in the development, manufacturing, and processing of materials and considerable efforts have been devoted to producing phase diagrams for many different materials and applications.^[^ ^1^ ^]^ However, the composition space of multi‐component functional materials takes the general form A_1 − *x* − *y* − *z* − *m* − *n*_B_*x*_C_*y*_D_*z*_E_*m*_F_*n*_, where A, B, C, D, E, F represent components, such as elements in an alloy, ceramic, or oxide. The complexity and number of possible phase diagrams increases exponentially with the number of components.^[^ ^2^ ^]^ The process of acquiring multi‐component phase diagrams experimentally which requires careful synthesis across compositions and property characterization measurements can be time and cost consuming.^[^ ^3^ ^]^ Therefore, efficiently constructing multi‐component phase diagrams with desired features in multi‐component systems remains an outstanding problem.

Computational tools, including ab initio calculations based on density functional theory (DFT) and thermodynamic calculations using CALPHAD (Calculation of Phase Diagram), can accelerate phase diagram construction.^[^ ^4^, ^5^, ^6^, ^7^ ^]^ These codes build models for the thermodynamic properties to fit experimental data. However, considerable computational costs are often involved in studying multi‐component systems, especially those containing four or more components, as information on relatively new systems is not available in already assembled thermodynamic databases.^[^ ^8^ ^]^

We present an approach that uses as input data a sampling of where the bulk phases are for known systems. The transition temperatures are not required but if available provide refined estimates of the phase boundaries of unknown systems. Our approach relies on the use of machine learning (ML) methods, which have recently been applied to problems in materials science to search for materials with targeted properties as well as to accelerate simulation codes.^[^ ^9^, ^10^, ^11^, ^12^, ^13^, ^14^, ^15^, ^16^, ^17^, ^18^, ^19^, ^20^, ^21^, ^22^, ^23^, ^24^, ^25^, ^26^, ^27^, ^28^, ^29^ ^]^ There have been also several studies that examined how ML can be used to establish phase diagrams, such as the studies of phase diagrams on the Ising model,^[^ ^30^ ^]^ Lennard‐Jones systems,^[^ ^31^ ^]^ the reconstruction of temperature versus voltage hysteresis loops for a relaxor system,^[^ ^32^ ^]^ and a surface phase diagram from electronic structure calculations for electrochemical catalysis in IrO_2_,^[^ ^33^ ^]^ which are limited to the studies of just one specific compound. Our approach is not limited to specific components or compounds, but applies to families of material systems containing thousands of compounds, such as the BaTiO_3_‐based ferroelectric ceramics or NiTi‐based shape memory alloys (SMAs). In the present study, we will show that these ML methods provide a powerful set of tools, especially for small experimental data sets, to construct multi‐component phase diagrams with desired features in multi‐component systems as they efficiently predict, assess, and scan a large number of possible candidates by eliminating redundancies and expensive operations.^[^ ^34^ ^]^ In addition, we incorporate efficient sampling methods to rapidly refine the ML predictions^[^ ^35^, ^36^, ^37^, ^38^ ^]^ to guide new measurements to reduce overall uncertainties so that phase diagrams can be iteratively refined in the fewest number of measurements within an active learning paradigm. This allows the algorithm to choose the data from which it learns so that it may learn more efficiently with less training data.

**Figure 1:**
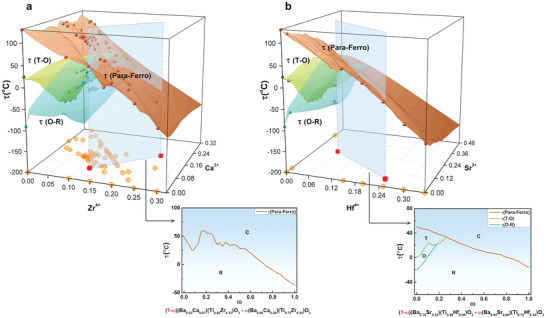
How we obtain pseudo‐binary temperature‐composition phase diagrams. To visualize in 3D space, a BaTiO_3_ ceramic system with only two compositional freedoms is shown. a) The training data as distributed on the three transition surfaces for the paraelectric to ferroelectric *τ*(Para‐Ferro), tetragonal to orthorhombic *τ*(T‐O), and orthorhombic to rhombohedral *τ*(O‐R) transitions. Machine learning models interpolate these surfaces to the whole space. b) A BaTiO_3_ ceramic system doped with Hf^4 +^ and Sr^2 +^ has no training data available for the co‐doped compositions, but machine learning models are able to predict the transition surface. As shown by the small panels on the right‐hand side, any two compounds in the bottom compositional plane can form a pseudo‐binary composition–temperature phase diagram, which can be estimated by machine learning models.

We will apply our approach to two families of materials systems containing thousands of compounds and multiple components, namely, the BaTiO_3_‐based ferroelectric ceramics and the NiTi‐based SMAs. A very large space of possibilities would be given by doping. BaTiO_3_ doped with Hf^4 +^, Zr^4 +^, and Sn^4 +^ at the Ti^4 +^ site, and Sr^2 +^, Cd^2 +^, and Ca^2 +^ at the Ba^2 +^ site gives rise to 10^13^ BaTiO_3_ possible binary phase diagram ceramics, as a phase diagram with at least one phase boundary can exist between any two solid solutions. Similarly, NiTi‐based alloys, in which Ti can be replaced by Hf, Zr, and Nb, and Ni by Fe, Cr and Co, can give rise to 10^14^ NiTi binary phase diagrams (Section S1, Supporting Information). Our objective will be to rapidly and efficiently construct any desired multi‐component phase diagram with targeted requirements within the multi‐dimensional composition space for these systems. **Figure** **1**a shows a BaTiO_3_ ceramic with only two compositional degrees of freedom, namely, Zr^4 +^ and Ca^2 +^ with the experimental training data distributed on three phase transition surfaces: paraelectric to ferroelectric *τ*(Para‐Ferro), tetragonal to orthorhombic *τ*(T‐O), and orthorhombic to rhombohedral *τ*(O‐R). Even when no training data are available for co‐doped compositions, that is, compositions containing both cations, as for BaTiO_3_ doped with Hf^4 +^ and Sr^2 +^ shown in Figure 1b, ML algorithms allow us to make predictions in the whole composition space, that is, including where data are not available. As shown by the cross‐sectional slices in the two panels in Figure 1, any two compounds with given values of the dopants can form a pseudo‐binary composition–temperature phase diagram which can be estimated by ML. The idea is general and can be extended to high‐dimensional phase diagrams involving many dopants.

Here we show how we can find two new multi‐component phase diagrams with given requirements by performing only three new experiments in each case. In particular, for the BaTiO_3_ ceramic we search for a compound with a phase diagram containing a triple point and a high transition temperature. This ensures a large temperature range for the ferroelectric phase, which is important for dielectric and piezoelectric applications. In fact, any combination of two compounds in the system, (Ba_1 − *x* − *y*_Sr_*x*_Ca_*y*_)(Ti_1 − *z* − *m* − *n*_Zr_*z*_Sn_*m*_Hf_*n*_)O_3_, can give rise to 12 795 pseudo‐binary phase diagrams. From these, we find that the particular phase diagram given by the two end compounds (1‐*ω*)(Ba_0.61_Ca_0.28_Sr_0.11_TiO_3_) and *ω*(BaTi_0.888_Zr_0.0616_Sn_0.0028_Hf_0.0476_O_3_), where *ω* is the mole fraction of an end compound of the binary phase diagram, fulfills the desired characteristics with a triple point and high transition temperature. Similarly, we show how to obtain a pseudo‐binary phase diagram for high temperature SMAs. Here the virtual space consists of 2.0× 10^7^ possible alloys of (Ti_1 − *x* − *y* − *z*_Hf_*x*_Zr_*y*_Nb_*z*_)_1 − *u*_(Ni_1 − *q* − *r* − *t*_Fe_*q*_Co_*r*_Cr_*t*_)_*u*_, and any combination of two alloys gives a pseudo‐binary phase diagram. Our predicted pseudo‐binary phase diagram combines (1‐*ω*)Ti_0.309_Ni_0.485_Hf_0.20_Zr_0.006_ with *ω*Ti_0.309_Ni_0.485_Hf_0.07_Zr_0.068_Nb_0.068_, with an austenite–martensite phase boundary. The alloy contains “Zr,” even though we do not have any alloy containing “Zr” in the training data. In addition, as most phase diagrams for solid solutions are out of equilibrium, our approach, relying as it does on well‐characterized experimental data, naturally incorporates the out of equilibrium metastable phases present in phase diagrams.

## 2 Results and Discussion

### 2.1 Design Strategy

**Figure 2:**
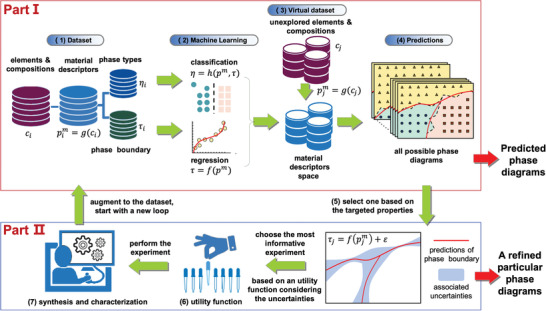
Flow chart of our design strategy containing two parts. Part I, the upper panel, predicts unexplored phase diagrams from surrogate models of classification and regression. Part II, the lower panel, optimizes a preselected phase diagram based on some targeted features *via* iterative experiments. The numbering refer to: (1) a database of compositions (*c*_*i*_), transition temperatures (*τ*_*i*_), and phases (*η*_*i*_). Each compound is uniquely described in terms of one or more material descriptors, (*p* ^*m*^), representing aspects of structure, chemistry and bonding. (2) Three approaches designed for phase diagram prediction. Approach 1a: classification learning to predict phases directly, *η* = *h*(*p* ^*m*^, *τ*); Approach 1b: regression on support vectors from classification; Approach 2: regression for each phase boundary to relate the transition temperature to the descriptors or features, *τ* = *f*(*p* ^*m*^) (3) For each unexplored composition, *c*_*j*_, that can include new elements, the material descriptors ($p_{j}^{m}$) are calculated using $p_{j}^{m}=g\left(c_{j}\right)$. (4) The machine learning models in (2) are directly applied to the unexplored composition space for given $p_{j}^{m}$. (5) A targeted phase diagram with uncertainties associated with the phase boundaries is selected. (6) Several utility functions utilizing the predictions and uncertainties are employed to select the next composition for (7) synthesis and characterization. The results from (7) augment the initial training data in (1).

**Figure** **2** shows our overall design strategy separated into two parts. Part I employs three machine learning strategies in the form of classification and regression tools to build surrogate models from data to predict and screen all unexplored phase diagrams. Part II optimizes the preselected phase diagram by iteratively guiding new synthesis of a specified compound with given composition and measurement of its transition temperature.

We thus 1) start with a database of known compositions (*c*_*i*_), transition temperatures (*τ*_*i*_), and phases (*η*_*i*_) as input to Part I. In order to generalize our machine learning models to elements absent in the training database, each compound is uniquely described in terms of one or more material descriptors, (*p* ^*m*^), representing aspects of structure, chemistry, and bonding. 2) We employ three approaches to estimate the phase boundaries. Approach 1a: We build a classification model to predict the phases from the descriptors and temperature (*τ*), *η* = *h*(*p* ^*m*^, *τ*), and the crossovers between different phases provide an estimate of the phase boundaries. Approach 1b: The support vectors from the classification performed in Approach 1 provide estimates of transition temperatures as they are at the margins separating the phases. These estimates can then be regressed to obtain a prediction of the full phase boundaries. Approach 2: As we have a data set on the transition temperatures themselves, a more precise regression model of these temperatures allows us to relate the transition temperatures to the descriptors, *τ* = *f*(*p* ^*m*^). 3) For each unexplored composition, *c*_*j*_, even involving new elements, the material descriptors ($p_{j}^{m}$) are calculated through $p_{j}^{m}=g\left(c_{j}\right)$. 4) The models built in (2) are directly applied to the unexplored composition space with known $p_{j}^{m}$ to obtain a prediction of the phase diagram. Thus, the output from Part I is an estimate of all possible phase diagrams from which the particular one with desired features is selected for Part II.

Part II guides new subsequent experiments to augment the data set so that the measured transition temperatures for predicted compositions are used to improve the model toward finding the optimized phase diagram. The emphasis here is to minimize the number of experiments so that the experimentally validated phase boundaries can be established rapidly. Hence, in 5) a desired phase diagram is selected and the uncertainties associated with the phase boundaries are estimated. Finally, in 6) the next composition is selected for 7) experimental synthesis and characterization. The experimental results are added to the initial training data in (1) and the loop repeats itself until the desired phase diagram with given accuracy is established.

### 2.2 Part I: Phase Diagram Prediction

#### 2.2.1 Data and Approaches

Our initial data set consists of 182 samples of ceramics and 130 samples of alloys compiled from measurements in our own laboratory. Each composition in the training data has one or more measured transition temperatures. From this data, we generate a data set of 3986 for ceramics and 2720 for alloys with given composition, temperature, and phase. This data includes points between the various transitions across the family of ceramics and alloys that we have studied experimentally. The details of these training data sets are given in Section S2, Supporting Information.

The material descriptors are calculated for each composition by a weighted fraction of elemental properties including radius, electronegativity, valence electron numbers, etc.^[^ ^24^, ^39^, ^40^ ^]^ Details of our down selection of descriptors for both systems are discussed in Section S3, Supporting Information, and the final selected material descriptors are listed in Tables 7– 9, Supporting Information.

We study two basic approaches, depending on whether we utilize transition temperature data or not. Approach 1 uses as input temperature, discretized into integer values for each composition, and the known phase, which serves as a label, corresponding to that temperature. Within this approach we employ two strategies. Approach 1a predicts directly the phase at any given temperature via ML models known as classifiers. The phase boundary positions are thus inferred from the change of phases with temperature. Although this approach does not require experimental transition temperatures, it requires adequate data to predict smooth and accurate boundaries. A related but more predictive approach using the same input, is Approach 1b which utilizes output from the classifier known as support vectors, which serve as estimates or surrogates for the phase boundary or transition temperatures between phases. We utilize these as input to a regression model to predict the margin or full phase boundary, including temperatures at points that did not have support vectors from the classification model.

In contrast to Approach 1, we make use of experimental transition temperatures in Approach 2 to construct a purely regression model based on Universal Kriging to predict transition temperatures everywhere, and thereby the full phase boundary.

#### 2.2.2 Validation

**Figure 3:**
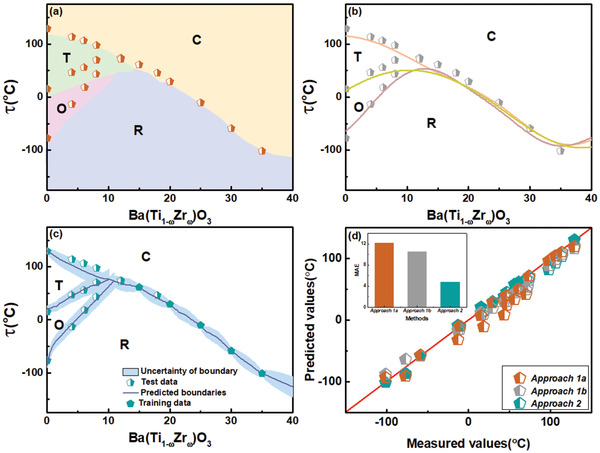
A comparison of the results for the Ba(Ti_1 − *ω*_Zr_*ω*_)O_3_ ferroelectric ceramic system using our three approaches. a) The underlying color coded phase diagram, which is the output from Approach 1a, is based on the results from an SVM classifier that predicts the phases as labels at any temperature. b) The phase boundaries obtained by using Approach 1b, which regresses the predicted support vectors from the classifier to predict the phase boundaries for different transitions shown by solid lines. Experimental transition temperatures are shown by pentagons. c) The predictions from Approach 2, based on the Universal Kriging model using experimental transition temperature data, are compared to experimental data from the literature. d) Diagonal plot for the predicted and reported values for the data from experiments in (a–c). Inset shows the mean absolute error(MAE) for each approach using the test and training data. The accuracy improves from Approach 1a to Approach 2.

We tested our approaches on two simpler systems, namely a Ba(Ti_1 − *ω*_Zr_*ω*_)O_3_ ferroelectric ceramic system and the alloy Ti_0.50_(Ni_0.50 − *ω*_Cu_*ω*_). We describe in detail the results for the BaTiO_3_‐based system in **Figure** **3** and give the results for the SMA, which uses the identical approach. Approach 1a predicts either cubic (C), tetragonal (T), orthorhombic (O), or rhombohedral (R) phases for every (*ω*, *τ*). Figure 3a shows the predicted Ba(Ti_1 − *ω*_Zr_*ω*_)O_3_ phase diagram from the classifier using 3986 training data points, where the phases are labeled and color coded. The half‐filled pentagons are the test data for transition temperatures from the literature and our own experiments.^[^ ^41^ ^]^ The crossover from one phase to another in the *ω*‐*τ* plane essentially provides an estimate of the phase boundaries C–T, T–O, O–R, and C–R. The large area above 0.95 in the Receiver Operating Curve (Section S4, Supporting Information), which is a plot of the true positive rate versus true negative rate for different classification thresholds, indicates an accurate and robust classification. The smoothness of the phase boundaries in Figure 3a is determined by the discretization or number of points used. Details of performance of the classifiers and the dependence of our results on data requirements are given in Section S4, Supporting Information, where we also compare the relative merits of different classifiers. The comparison to the experimental test data in Figure 3a shows that the approach does not capture the O–T and O–R phase boundaries or the location of the triple point, other than the Para‐to‐Ferro phase boundary (C–T, C–R).

The results from the more predictive Approach 1b using classification, followed by regression, are shown by the curves in Figure 3b and details are given in Section S5, Supporting Information. The same input as before is used for the classification, and the predicted support vectors that serve as estimates of transition temperatures separating two phases are used as input to the regression to predict a full phase boundary (solid lines shown in Figure 3b). This approach better approximates the O–R and T–O transition lines and the position of the triple point compared to the direct classification Approach 1a. Thus, the regression following the classification leads to considerable improvement.

Finally, we study via Approach 2 how well regression will perform on the experimental transition data itself rather than using the support vectors from classification. We therefore trained a Kriging model for all three transitions using our full training database of 182 ceramics in which all samples have a Para‐to‐Ferro transition but subsets have also a T–O transition as well all three transitions. The Kriging models give the CV_e*rror*_ in the transition temperatures of ≈ 4.40% for the Para‐to‐Ferro transition, ≈ 5.92% for T–O, and ≈ 8.04% for O–R. Details of the model performance are shown in Section S5, Supporting Information. The solid lines of Figure 3c are predictions and their uncertainties associated with the boundaries from the Kriging model. The predictions for *τ*(Para‐Ferro) are quite consistent with our experimental data. For the T–O and O–R phase boundaries, even though there are no training data for the phase diagram, we can still estimate the phase boundaries and compare the predictions with transition data from the literature.^[^ ^41^ ^]^

The accuracy of the predictions from the three approaches for all the three transitions combined are compared in the diagonal plot of Figure 3d, where we show how the mean absolute error (MAE) decreases significantly from Approach 1a to Approach 2. The Kriging models of Approach 2 using experimental transition temperatures faithfully model the phase boundaries and the position of the triple point compared to direct classification (Approach 1a) or the use of regression on predicted support vectors from classification (Approach 1b).

**Figure 4:**
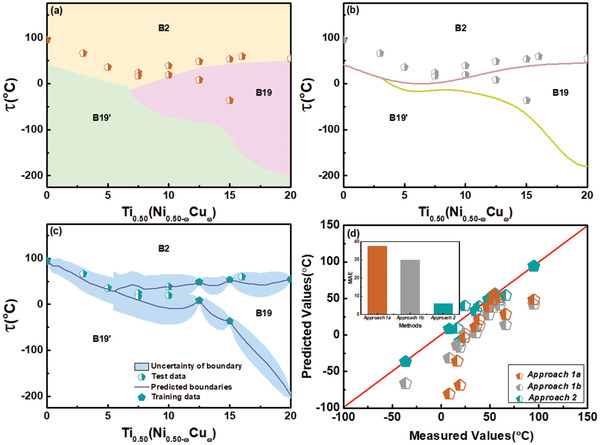
A comparison of the results for the SMA system Ti_0.50_(Ni_0.50 − *ω*_Cu_*ω*_) using the three approaches. a) The underlying color coded phase diagram, which is the output from Approach 1a, based on the results from an SVM classifier that predicts the phases as labels at any temperature. b) The phase boundaries shown by solid lines obtained with Approach 1b using the predicted support vectors from the classifier in a regression model. Experimental transition temperatures are shown by half pentagons. c) The predictions from Approach 2, based on the Universal Kriging model using experimental transition temperature data, are compared with experimental data from the literature. d) Diagonal plot for the predicted and reported values for the data from experiments in (a–c). Inset shows the mean absolute error (MAE) for each approach using the test and training data. The accuracy improves from Approach 1a to Approach 2.

**Figure** **4**a–c shows our predictions for Ti_0.50_(Ni_0.50 − *ω*_Cu_*ω*_) alloys from Approach 1a using a classifier, Approach 1b using classification and regression, and Approach 2 using Kriging. The classifier has an accuracy of ≈0.98 (Section S4, Supporting Information) and there are two phase boundaries separating the B2 austenite and B19 and B19' martensite phases. The experimental transition data shown in Figure 4a–c are from the literature,^[^ ^42^ ^]^ and our synthesis of compounds with composition *ω*= 3, 16 in our laboratory. This formed the test data for the classification, and for the regression in Approach 2 the half pentagons in Figure 4c formed the test data and the solid pentagons were used for training. The predicted versus measured values using all the test and training data are shown in the diagonal plot in Figure 4d. The mean absolute error (MAE) again decreases from Approach 1a to Approach 1b to Approach 2. Compared to Approach 2 for both ceramic and SMA, Approaches 1a, 1b do not perform as well. The likely reason for this is that the support vectors of the hyperspace, after being projected to a specific phase diagram, do not lie on the true margin or boundary of the two phases. Also, the experimental transition data used in Approach 2 appears far more informative, and for Ti_0.50_(Ni_0.50 − *ω*_Cu_*ω*_), Approaches 1a, 1b compared to the ceramic suffer from limited training data, especially for the martensite transition. Therefore, Approach 2 is chosen for subsequent phase diagram prediction and optimization.

#### 2.2.3 Phase Diagrams with Desired Characteristics

**Figure 5:**
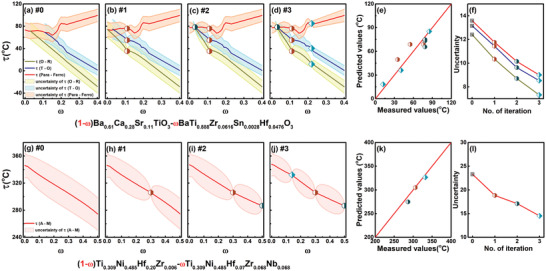
The efficient validation and optimization of preselected phase diagrams for a) (1‐*ω*)Ba_0.61_Ca_0.28_Sr_0.11_TiO_3_‐*ω*BaTi_0.888_Zr_0.0616_Sn_0.0028_Hf_0.0476_ and g) (1‐*ω*)Ti_0.309_Ni_0.485_Hf_0.20_Zr_0.006_‐*ω*Ti_0.309_Ni_0.485_Hf_0.07_Zr_0.068_Nb_0.068_. b–d) The phase diagrams obtained from subsequent iterations for the ferroelectric ceramic and h–j) are for the SMA system. In each iteration, a particular composition selected by “Maximum Variance” is synthesized and measured. The results augment the data set and a new iteration resumes. e,k) show that the predictions of phase boundaries are consistent with the measured values for each iteration, as the points reasonably follow a diagonal line. f,l) show the overall decrease in uncertainty associated with the phase boundary.

For the BaTiO_3_‐based system, with a design criteria of a triple point and high transition temperature, we can in principle dope at both the A and B sites, which would give a very large space of possibilities. We thus constrain the space by considering separately dopants at the A and B sites, and then construct the phase diagram that includes both A and B dopants. For the B site, we consider the system (1‐*ω*)BaTiO_3_‐*ω*Ba(Ti_1 − *x* − *y* − *z*_Zr_*x*_Sn_*y*_Hf_*z*_)O_3_ for which there are 11680 phase diagrams, and our aim is to find a solid solution with only a C to R transition with a transition temperature as high as possible. We find that approximately 200 phase diagrams meet this requirement within a certain error range. Amongst them, the system (1‐*ω*)BaTiO_3_ ‐ *ω*Ba(Ti_0.6_Zr_0.22_Sn_0.01_Hf_0.17_)O_3_ has multiple transitions for *ω* < 0.27 but only a C–R transition for *ω* > 0.27. The predicted phase diagram with a triple point at *ω* = 0.27 of 77.58 °C is shown in Section S6, Supporting Information. We thus choose a composition with *ω*=0.28, that is, Ba(Ti_0.888_Zr_0.0616_Sn_0.0028_Hf_0.0476_)O_3_ as the R end with a transition temperature of 73.18 °C, which is a relatively high C to R transition temperaturecompared to compounds studied in the literature such as Ba(Ti_0.8_Zr_0.2_)O_3_‐(Ba_0.7_Ca_0.3_)TiO_3_ with a triple point of 57°C.^[^ ^43^ ^]^Similarly, we search for a T end with a C to T transition accompanied by the largest value in the temperature *δ*(*Tc* − *Tt*), where *Tt* is the tetragonal to orthorhombic transition temperature. We scanned the 1115 possible phase diagrams in the system: (1‐*ω*)(Ba_1 − *v* − *u*_Ca_*v*_Sr_*u*_)TiO_3_‐*ω*BaTiO_3_. This led us to select the phase diagram for (1‐*ω*)(Ba_0.61_Ca_0.28_Sr_0.11_)TiO_3_‐*ω*BaTiO_3_ with a composition *ω* = 0 chosen for the T end with the largest *δ*(*Tc* − *Tt*) = 199.47°C. By combining the preselected T end and R ends, we form a new phase diagram with the predicted phase boundaries shown in **Figure** **5**a that meets our criterion. Often it is difficult other than by trial and error to know which phase diagram needs to be established as the search space is large. Our method is able to address this problem and go beyond what has so far been possible in the BaTiO_3_‐based system.

Similarly in the search for high temperature SMAs, our virtual space consists of alloys in the family (Ti_1 − *x* − *y* − *z*_Hf_*x*_Zr_*y*_Nb_*z*_)_1 − *u*_(Ni_1 − *q* − *r* − *t*_Fe_*q*_Co_*r*_Cr_*t*_)_*u*_, such that any combination of two alloys in the space of alloys gives a pseudo‐binary phase diagram. By scanning the transition temperatures of all the alloys, we find that Ti_0.309_Ni_0.485_Hf_0.20_Zr_0.006_ has the largest predicted phase transition temperature of 346.15 °C as one terminal of the solid solution. For the other terminal, we choose the alloy Ti_0.309_Ni_0.485_Hf_0.07_Zr_0.068_Nb_0.068_ as it has a high transition temperature >100 °C and a high entropy of mixing of Δ*S*_mix_=10.5221 (i.e., given that the dopants Hf, Zr, and Nb are in equal mole fraction). The predicted pseudo‐binary phase diagram with an austenite–martensite phase boundary is given by the system: (1‐*ω*)Ti_0.309_Ni_0.485_Hf_0.20_Zr_0.006_‐*ω*Ti_0.309_Ni_0.485_Hf_0.07_Zr_0.068_Nb_0.068_, shown in Figure 5e. We note that this new SMA phase diagram contains the element “Zr,” which is not presented in our training database, showing our approach extends to unexplored spaces.

### 2.3 Part II: Optimization of the Predicted Phase Diagram

Our machine learning predictions so far inevitably contain uncertainties and therefore the validation and optimization of the predicted phase diagrams through experiments is a crucial element of our approach (Part II of Figure 2) that we now address (Experimental details in Section S7, Supporting Information). The problem is how to choose optimal compositions so that the number of new experiments can be minimized. We have demonstrated previously that an optimal strategy for selecting the compositions for the next experiment is to choose the predictions from the ML with the largest variance.^[^ ^44^ ^]^

Hence, with the predicted phase diagram for the ferroelectric in Figure 5a as a starting point, we chose compositions for synthesis that gave the largest predicted variance ($V_{\omega }$). For compositions undergoing several transitions (*ω* > 0.1 in Figure 5a), the variance $V_{\omega }$ is calculated as an average value of the variance for each phase boundary. As *ω* = 0.1 has the largest variance, we chose the compound with *ω* = 0.1 as the first candidate and synthesized and characterized it to obtain the transition temperatures and augmented the initial training data set. The regression models for the boundaries are retrained and the predicted phase boundaries modified by the newly added experimental data, as shown in Figure 5b. Importantly, the associated uncertainties are altered, for example, the compositions adjacent to the experimental data now have lower uncertainties. This allows us to choose a composition away from the existing data according to maximum variance. This iteration loop was executed three times and the results from the second and third iterations are shown in Figure 5c and Figure 5d, respectively, with the validated and optimized final phase diagram in Figure 5d. The predictions of the phase boundaries are quite consistent with the measured values for each iteration, as in Figure 5e. By iteratively augmenting new data selected by the utility function, the overall uncertainties in the phase boundary prediction decrease (Figure 5f). Three new experiments were adequate to essentially establish the final phase diagram.

We similarly carried out the design loop three times to validate and optimize the new SMA phase diagram we obtained in Figure 5g. The phase diagrams for subsequent iterations are shown in Figure 5h–j and Figure 5k shows the consistency of the predicted values with measured values. As for the BaTiO_3_ example, the overall uncertainty associated with the phase boundary decreases with iterations, as Figure 5l shows. Thus, optimization algorithms are important in accelerating the construction of validated phase diagrams.

### 2.4 Discussion

The prediction and validation of multi‐component phase diagrams containing a combination of several elements remains a widely studied and outstanding problem in materials science. As the phase space is large, we have in this work demonstrated a strategy for families of materials systems that can be doped with different elements or components that utilizes existing experimental data. The idea is to build a high‐dimensional virtual space of all possible phase diagrams involving these dopants or components and their mole fractions, and then screen it to construct particular multi‐component phase diagrams with given design criteria, such as high transition temperatures, triple, or critical points. Phase diagrams containing elements not present in the training database can be predicted, as we have demonstrated with our SMA example. In addition, as we start with experimental data, our approach naturally includes both equilibrium and metastable phases. Beyond prediction, a key aspect of this work is also experimental refinement and optimization of the initial phase diagram. Here we show how methods from experimental design, particularly those related to minimizing overall uncertainty in an active learning paradigm with feedback from experiments, allow us to minimize the number of experiments needed to three in our examples.

The assumption underlying our approach is embodied in the initial input database we have assembled that contains information about the phases (or phase diagram where available) of over 182 solid solutions with varying dopants. This includes bulk phases at given temperatures and/or the transition temperatures themselves. The latter, where available, also provide data of the phases themselves. Thus, we are able to use supervised ML methods, such as classification and regression, to make predictions of desired phase diagrams. We have thus assumed that phase diagrams of ceramics and alloys are sufficiently smooth so that a relatively small sample in temperature‐composition space is adequate to interpolate from in order to predict a phase boundary or surface. In both of our examples, we find that regression using transition temperature data performs better in capturing phase boundaries than classification using phase and temperature data. However, our approach does not allow us to predict any new phases, as studied in certain cases using unsupervised learning methods.^[^ ^30^ ^]^

## 3 Conclusion

The two new binary phase diagrams we have obtained illustrate how our approach can be used for targeted design. The complexity of these solid solutions is beyond any computational method available to date. We have predicted the ferroelectric ceramic (1‐*ω*)(Ba_0.61_Ca_0.28_Sr_0.11_TiO_3_) ‐*ω*(BaTi_0.888_Zr_0.0616_Sn_0.0028_Hf_0.0476_O_3_) with a relatively high transition temperature and triple point for applications which often demand a large d_33_, dielectric constant, vertical morphotropic phase boundary, or superior electrocaloric properties. Similarly, the NiTi‐based alloy (1‐*ω*)(Ti_0.309_Ni_0.485_Hf_0.20_Zr_0.006_) ‐*ω*(Ti_0.309_Ni_0.485_Hf_0.07_Zr_0.068_Nb_0.068_) containing Zr is designed to have a high transition temperature. We have been able to find this even without any Zr containing solid solutions in our training data. The same strategy can be applied to find alloys with prescribed requirements, such as those within the family of magnesium alloys or high entropy alloys (HEA). Although we have focused on experimental databases and performed experiments for validation, our approach is directly portable to computational problems and data. For example, in electrochemical analysis applications the compound with a given desired surface phase diagram can be predicted from a database containing the results of electronic structure calculations from a large number of compounds.^[^ ^33^ ^]^ Thus, due to the low computational costs, our approach provides a recipe to rapidly predict and scan a high‐dimensional virtual space of phase and temperature information for different compounds for targeted design, especially where the training data is relatively small.

## 4 Experimental Section

##### Experimental Methods—Preparation and Characterization of BaTiO_3_‐Based Ceramics

The ferroelectric ceramics were prepared by a conventional solid‐state reaction method with the starting materials of BaCO_3_ (99.8%), CaCO_3_ (99.9%), SrCO_3_ (99.9%), BaZrO_3_ (99.9%), SnO_2_ (99.9%), HfO_2_(99.8%), and TiO_2_ (99.6%). The calcination was performed at 1350 ^*o*^C for 3 h and sintering was done at 1450 ^*o*^C for 3 h in air. All the samples were synthesized under the same conditions to reduce the dependence of targeted property on processing. The sintered samples for dielectric measurements were polished to obtain parallel sides and coated with silver electrodes. The transition temperatures were determined by the temperature dependence of dielectric permittivity.

##### Preparation and Characterization of Shape Memory Alloys

The base ingot for the SMA was made by arc melting from pure Ti (99.995%), Ni (99.995%), Hf (99.99%), Zr (99.99%), and Nb (99.5%) in an argon atmosphere. The specimens for measurements were spark‐cut from the ingot and then solution treated at 1273 K for 1 h in an evacuated quartz tube, followed by water quenching. Differential scanning calorimetry (DSC) measurements were performed with a cooling/heating rate of 10 K min^−1^ to determine the martensitic transformation temperatures using endothermic peak values.

##### Statistical Inference and Analysis—Feature Selection

Feature selection methods, such as Gradient Boosting, Pearson Map, and Best subset with a Gaussian kernel to down select the most relevant materials descriptors (Section S3, Supporting Information) were utilized.

##### Statistical Inference and Analysis—Regressors and Classifiers

Several machine learning models were trained for the three Approaches.

- Approach 1a: Support Vector Machine (SVM) and Random Forest for classification.
- Approach 1b: Support Vector Machine for classification and Support Vector Regressor (SVR) for regression.^[^ ^45^ ^]^
- Approach 2: Kriging model for regression.

The Kriging model^[^ ^46^ ^]^ was employed for prediction and to estimate uncertainties. The *DiceKriging* package implementation of the machine learning models in the statistical studio R provided by Roustant et al. was utilized.^[^ ^47^ ^]^

##### Statistical Inference and Analysis—Bayesian Optimization

Bayesian optimization was extensively used for experimental design in choosing the next candidates.

##### Statistical Inference and Analysis—Data and Analysis

The data sets were compiled from the results of the previous laboratory experiments. All experiments were performed under the same controlled conditions and protocols to minimize variability. The few data points that were used from the literature were for the purposes of validating the predictions. The laboratory data consisted of 182 ceramic samples and 130 SMAs, which formed the training data (Section S2, Supporting Information). The preprocessing and scaling of the data were performed in the standard manner using studio R. The experimental measurements do suffer from noise. In our analysis it is assumed that the noise obeys a Gaussian distribution, $\varepsilon ∼N(0,\tau^{2})$, and set *τ* ^2^ to 10. The noise was considered in the modeling process.

## Conflict of Interest

The authors declare no conflict of interest.

## Supporting information

Supporting Information
