# Supplementary material for: Determining Multi‐Component Phase Diagrams with Desired Characteristics Using Active Learning
Source: Adv Sci (Weinh). 2020 Nov 23;8(1):2003165. doi: 10.1002/advs.202003165 (PMC7788591; doi:10.1002/advs.202003165)
Supplement: Supplementary file 1 — Supporting Information [file ADVS-8-2003165-s001.pdf]

© 2020 Wiley-VCH GmbH

ADVANCED SCIENCE
Open Access

Supporting Information

for *Adv. Sci.,* DOI: 10.1002/advs.202003165

Determining Multi-Component Phase Diagrams with Desired Characteristics Using Active Learning

Yuan Tian, Ruihao Yuan, Dezhen Xue, Doctor, Yumei Zhou,* Yunfan Wang, Xiangdong Ding, Jun Sun, and Turab Lookman**

Supplementary document for
Determining Multi-component Phase Diagrams with Desired Characteristics using Active Learning

Yuan Tian,[1] Ruihao Yuan,[1] Dezhen Xue,[1,*] Yumei Zhou,[1,†] Yunfan Wang,[1] Xiangdong Ding,[1] Jun Sun,[1] and Turab Lookman[2,‡]

[1] *State Key Laboratory for Mechanical Behavior of Materials, Xi'an Jiaotong University, Xi'an 710049, China.*
[2] *Theoretical Division, Los Alamos National Laboratory, Los Alamos, New Mexico 87545, USA.*
(Dated: August 18, 2020)

S1. SEARCH SPACE

A. Definition of pseudo-binary phase diagrams in shape memory alloys

Supplementary Figure 1 illustrates how we define pseudo-binary phase diagrams in NiTi shape memory alloys. For the purposes of visualization, we choose simpler systems with two different dopants. Machine learning algorithms are able to interpolate the phase transition surface to the whole composition space. As shown by the vertical plans and small panels in Supplementary Figure 1, any pseudo-binary composition-temperature phase diagram between any two compounds can be estimated, no matter whether the training data are available for the co-doped compositions or not. The idea is general and can be extended to high dimensional spaces with multiple components.

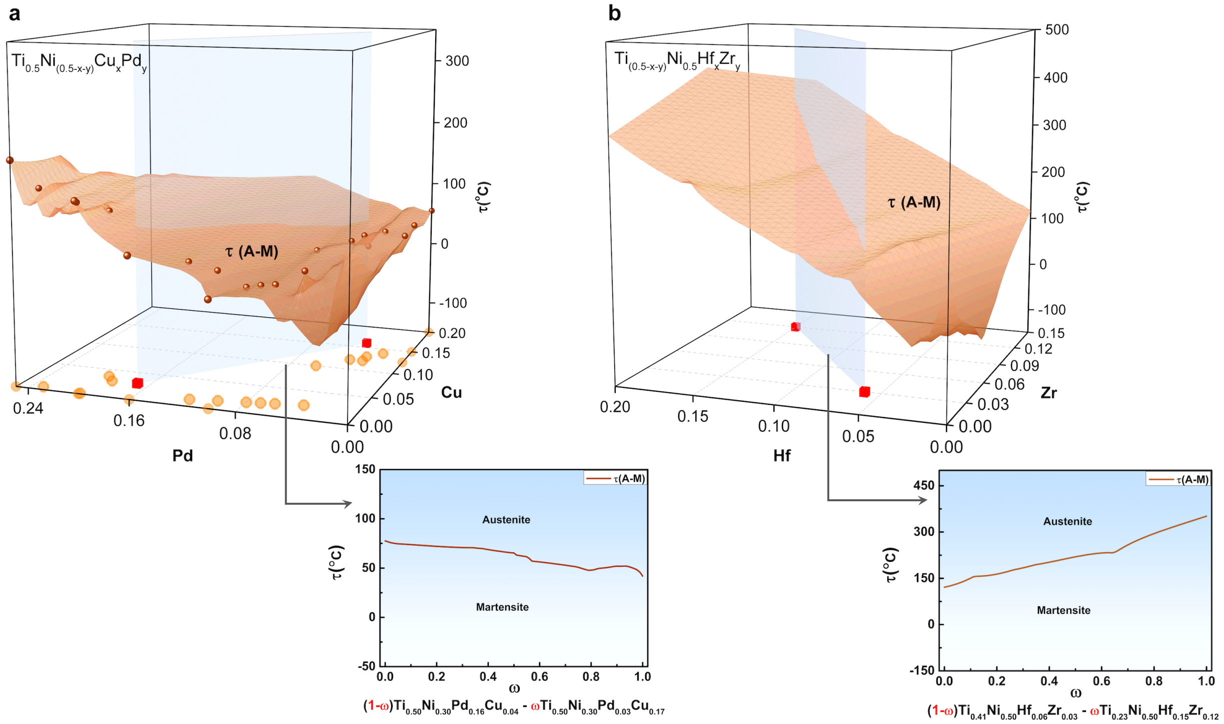

Supplementary Figure 1 | The illustration of our idea to obtain pseudo-binary temperature-composition phase diagrams. To visualize in 3D space, a NiTi based shape memory alloy system with only two compositional degrees of freedom is shown. (a) the training data are distributed on the austenite(A) to martensite(M) transition surface. Machine learning allows us to interpolate the surface throughtout the whole space. (b) The space of NiTi based shape memory alloys doped with Hf and Zr with no available training data for co-doped compositions. However, machine learning provides an initial prediction across the whole transition surface. As shown by the small panels, the projection of the surface in the bottom compositional plane can give rise to two end compounds that form a pseudo-binary composition-temperature phase diagram, which can be estimated by machine learning models.

B. $BaTiO_3$ based solid solutions

In the present work, we are interested in the phase diagram of (1-ω)$BaTiO_3$-$\omega$$Ba(Ti_{1-x-y-z}Zr_xSn_yHf_z)O_3$ and (1-ω)$(Ba_{1-v-u}Ca_vSr_u)TiO_3$-$\omega$$BaTiO_3$, where w is the mole fraction of one end of the pseudo-binary phase diagram. The constraints include $0 \leq x, y, z \leq 0.30, x+y+z \leq 0.40, 0 \leq v, u \leq 0.30, v+u \leq 0.40$, where x, y, z, v, u vary in steps of 0.01. As a result, the former family includes 11,680 different phase diagrams and the latter 1,115 phase diagrams.

For each phase diagram, ω is discretized into 100 values within the range from 0 to 1. Thus, there are 101 compounds in each phase diagram. We chose a $(Ba_{0.61}Ca_{0.28}Sr_{0.11})TiO_3$ compound from the latter family and a $Ba(Ti_{0.888}Zr_{0.0616}Sn_{0.0028}Hf_{0.0476})O_3$ compound from the former family to form a new phase diagram with specific requirements.

In principle, any combination of the compounds can form a pseudo-binary phase diagram. There exist 8.8×10^6 compounds in the solid solution of $(Ba_{1-x-y}Sr_xCa_y)(Ti_{1-z-m-n}Zr_zSn_mHf_n)O_3$ with the above constraints. In total there are $C^2_{8.8\times10^6} = 3.8\times10^{13}$ possible phase diagrams which can be estimated.

C. NiTi based shape memory alloys

The system we considered is $(Ti_{1-x-y-z}Hf_xZr_yNb_z)_{1-u}(Ni_{1-q-r-t}Fe_qCo_rCr_t)_u$. The constraints include $0.485 \leq u \leq 0.5$, $0 \leq x, y \leq 0.20$, $0 \leq z \leq 0.1$, $x+y+z \leq 0.25$,and $0 \leq q, r, t \leq 0.05$, and $q+r+t \leq 0.05$. The x, y, z, q, r, t can vary in steps of 0.005. In total there exist 2.0×10^7 possible compounds. Any combination of the compounds can form a pseudo-binary phase diagram. There are $C^2_{2.0\times10^7} = 2.1\times10^{14}$ possible phase diagrams.

S2. TRAINING DATABASE

Our initial training dataset consists of compositions and transition temperature, including 182 samples of ceramics and 130 samples of alloys assembled from our own laboratory.

A. Training database of $BaTiO_3$ based solid solutions

1. *Training data for Approach 2*

The training data using the Universal Kriging model of *Approach 2* contains the compositions of elements in the ferroelectric systems. The ferroelectric ceramics undergo transitions from the paraelectric to ferroelectric phase, ferroelectric tetragonal to orthorhombic phase, and ferroelectric orthorhombic to rhombohedral phase. Thus, our training data contains three transition temperatures as the target quantities, that is τ(Para-Ferro), τ(T-O) and τ(O-R). Some compounds may not have all three transitions and the absent transition temperature are set to *NA.* in our training data. The first 15 data in the database are shown in Supplementary Table 1 below as an example.

Supplementary Table 1 | The Universal Kriging regression training dataset for ferroelectric ceramics with concentrations of elements and targeted transition temperatures. Only the first 15 observations are shown.

Ba	Ca	Sr	Cd	Ti	Zr	Sn	Hf	τ(Para-Ferro)	τ(T-O)	τ(O-R)
0.9500	0.0000	0.0000	0.0500	1.0000	0.0000	0.0000	0.0000	125.43	15.55	-68.57
0.7500	0.0000	0.0000	0.2500	1.0000	0.0000	0.0000	0.0000	123.86	31.20	-67.31
0.9650	0.0350	0.0000	0.0000	0.7750	0.2250	0.0000	0.0000	7.45	*NA.*	*NA.*
0.8425	0.1575	0.0000	0.0000	0.8625	0.1375	0.0000	0.0000	58.21	*NA.*	*NA.*
1.0000	0.0000	0.0000	0.0000	0.9900	0.0000	0.0100	0.0000	120.20	24.48	-52.60
1.0000	0.0000	0.0000	0.0000	0.9300	0.0000	0.0700	0.0000	73.86	42.86	16.87
0.9700	0.0300	0.0000	0.0000	0.8920	0.0000	0.1080	0.0000	49.82	38.34	29.18
0.9100	0.0900	0.0000	0.0000	0.9160	0.0000	0.0840	0.0000	72.90	22.78	-6.2
0.8500	0.1500	0.0000	0.0000	0.9000	0.1000	0.0000	0.0000	94.61	29.5	-1.07
0.8560	0.1440	0.0000	0.0000	0.9400	0.0600	0.0000	0.0000	97.93	7.61	-29.64
0.9500	0.0000	0.0500	0.0000	0.8000	0.2000	0.0000	0.0000	29.43	*NA.*	*NA.*
0.8300	0.0000	0.1700	0.0000	0.7900	0.2100	0.0000	0.0000	1.51	*NA.*	*NA.*
0.8800	0.1200	0.0000	0.0000	0.8880	0.0000	0.1120	0.0000	48.67	-5.54	-30.22
0.8650	0.1350	0.0000	0.0000	0.8810	0.0000	0.1190	0.0000	27.19	7.52	-7.31
0.9300	0.0700	0.0000	0.0000	0.9700	0.0000	0.0200	0.0100	97.97	-2.78	-62.26

2. *Training data for Approach 1a and Approach 1b*

For classification for both *Approach 1a* and *Approach 1b*, the temperature (τ) is discretized into several integer values for a given composition. Within each temperature range between transition temperatures, the composition is given the label of the particular phase. With $BaTiO_3$ as an example, $BaTiO_3$ undergoes in total three transitions from C to T to O to R with cooling. The three transition temperatures are 129.85^{o}C, 24.85^{o}C, -90.15^{o}C. We give the phase of $BaTiO_3$ above 129.85^{o}C the label C, the phase between 129.85^{o}C and 24.85^{o}C the label T, the phase between 24.85^{o}C and -90.15^{o}C the label O and the phase below -90.15^{o}C the label R. A database for classification containing 3986 labeled data is thus established. The first 30 lines of data in the database are shown in Supplementary Table 2 below as an example.

Supplementary Table 2 | The classification training dataset for ferroelectric ceramics includes the concentrations of elements, temperatures and labels of phases. Only the first 30 lines.

Ba	Ca	Sr	Ti	Cd	Zr	Sn	Hf	τ	η
1.0000	0.0000	0.0000	1.0000	0.0000	0.0000	0.0000	0.0000	179.85	C
1.0000	0.0000	0.0000	1.0000	0.0000	0.0000	0.0000	0.0000	169.85	C
1.0000	0.0000	0.0000	1.0000	0.0000	0.0000	0.0000	0.0000	159.85	C
1.0000	0.0000	0.0000	1.0000	0.0000	0.0000	0.0000	0.0000	149.85	C
1.0000	0.0000	0.0000	1.0000	0.0000	0.0000	0.0000	0.0000	139.85	C
1.0000	0.0000	0.0000	1.0000	0.0000	0.0000	0.0000	0.0000	129.85	T
1.0000	0.0000	0.0000	1.0000	0.0000	0.0000	0.0000	0.0000	119.85	T
1.0000	0.0000	0.0000	1.0000	0.0000	0.0000	0.0000	0.0000	109.85	T
1.0000	0.0000	0.0000	1.0000	0.0000	0.0000	0.0000	0.0000	99.85	T
1.0000	0.0000	0.0000	1.0000	0.0000	0.0000	0.0000	0.0000	89.85	T
1.0000	0.0000	0.0000	1.0000	0.0000	0.0000	0.0000	0.0000	74.85	T
1.0000	0.0000	0.0000	1.0000	0.0000	0.0000	0.0000	0.0000	64.85	T
1.0000	0.0000	0.0000	1.0000	0.0000	0.0000	0.0000	0.0000	54.85	T
1.0000	0.0000	0.0000	1.0000	0.0000	0.0000	0.0000	0.0000	44.85	T
1.0000	0.0000	0.0000	1.0000	0.0000	0.0000	0.0000	0.0000	34.85	T
1.0000	0.0000	0.0000	1.0000	0.0000	0.0000	0.0000	0.0000	14.85	O
1.0000	0.0000	0.0000	1.0000	0.0000	0.0000	0.0000	0.0000	4.85	O
1.0000	0.0000	0.0000	1.0000	0.0000	0.0000	0.0000	0.0000	-5.15	O
1.0000	0.0000	0.0000	1.0000	0.0000	0.0000	0.0000	0.0000	-15.15	O
1.0000	0.0000	0.0000	1.0000	0.0000	0.0000	0.0000	0.0000	-25.15	O
1.0000	0.0000	0.0000	1.0000	0.0000	0.0000	0.0000	0.0000	-80.15	O
1.0000	0.0000	0.0000	1.0000	0.0000	0.0000	0.0000	0.0000	-70.15	O
1.0000	0.0000	0.0000	1.0000	0.0000	0.0000	0.0000	0.0000	-60.15	O
1.0000	0.0000	0.0000	1.0000	0.0000	0.0000	0.0000	0.0000	-50.15	O
1.0000	0.0000	0.0000	1.0000	0.0000	0.0000	0.0000	0.0000	-40.15	O
1.0000	0.0000	0.0000	1.0000	0.0000	0.0000	0.0000	0.0000	-100.15	R
1.0000	0.0000	0.0000	1.0000	0.0000	0.0000	0.0000	0.0000	-110.15	R
1.0000	0.0000	0.0000	1.0000	0.0000	0.0000	0.0000	0.0000	-120.15	R
1.0000	0.0000	0.0000	1.0000	0.0000	0.0000	0.0000	0.0000	-130.15	R
1.0000	0.0000	0.0000	1.0000	0.0000	0.0000	0.0000	0.0000	-140.15	R

B. Training data for NiTi-based shape memory alloys

1. *Training data for Approach 2*

The training data used for Universal Kriging regression in *Approach 2* contains the concentration of elements and targeted transition temperatures (τ(A-M) and τ(M1-M2)). The first 15 lines of data in the database are shown in Supplementary Table 3 below as an example.

2. *Training data for Approach 1a and Approach 1b*

Similar to the case of ferroelectric ceramics, the temperature (τ) is discretized into several integer values for each composition. Within each temperature range between transition temperatures, the composition is given a label for the particular phase. For example, $Ti_{0.50}Ni_{0.50}$ undergoes a transition from austenite to martensite at 95°C. We label the phase of $Ti_{0.50}Ni_{0.50}$ above 95°C as A and the phase below 95°C as M. A database for classification containing 2720 labeled data is thus established. The first 15 pieces of data in the database are shown in Supplementary Table 4 as an example.

Supplementary Table 3 | The Universal Kriging regression training dataset for shape memory alloys with concentrations of elements and targeted transition temperatures. Only the first 15 observations are shown as an example.

Ti	Ni	Cu	Fe	Pd	Co	Mn	Cr	Nb	Hf	τ(M1-M2)	τ(A-M)
0.5000	0.1000	0.0000	0.0700	0.3300	0.0000	0.0000	0.0000	0.0000	0.0000	$NA.$	92.57
0.5000	0.4105	0.0195	0.0000	0.0700	0.0000	0.0000	0.0000	0.0000	0.0000	$NA.$	19.56
0.5000	0.4000	0.0000	0.0000	0.1000	0.0000	0.0000	0.0000	0.0000	0.0000	$NA.$	5.77
0.5000	0.4870	0.0000	0.0130	0.0000	0.0000	0.0000	0.0000	0.0000	0.0000	$NA.$	37.74
0.5000	0.3000	0.0010	0.0000	0.1990	0.0000	0.0000	0.0000	0.0000	0.0000	$NA.$	128.14
0.5000	0.2980	0.0010	0.0010	0.2000	0.0000	0.0000	0.0000	0.0000	0.0000	$NA.$	113.17
0.5000	0.0000	0.0000	0.0000	0.4580	0.0000	0.0000	0.0420	0.0000	0.0000	$NA.$	336.46
0.5000	0.2500	0.0000	0.0130	0.2370	0.0000	0.0000	0.0000	0.0000	0.0000	79.42	99.25
0.5000	0.4800	0.0000	0.0000	0.0000	0.0000	0.0200	0.0000	0.0000	0.0000	-26.89	9.90
0.5000	0.4600	0.0000	0.0000	0.0000	0.0400	0.0000	0.0000	0.0000	0.0000	-37.29	-16.50
0.3500	0.5000	0.0000	0.0000	0.0000	0.0000	0.0000	0.0000	0.0000	0.1500	$NA.$	254.00
0.3500	0.4700	0.0300	0.0000	0.0000	0.0000	0.0000	0.0000	0.0000	0.1500	$NA.$	190.00
0.4850	0.4738	0.0000	0.0000	0.0136	0.0000	0.0000	0.6000	0.0216	0.0000	$NA.$	-3.14
0.4900	0.4488	0.0000	0.0054	0.0198	0.0000	0.0000	0.0000	0.0360	0.0000	$NA.$	13.25
0.5000	0.4600	0.0000	0.0000	0.0000	0.0000	0.0400	0.0000	0.0000	0.0000	$NA.$	-53.50

Supplementary Table 4 | The classification training dataset for shape memory alloys includes the concentrations of elements, temperatures and labels of phases. Only the first 10 lines are shown as an example.

Ti	Ni	Cu	Fe	Pd	Co	Mn	Cr	Nb	Hf	τ	η
0.5000	0.5000	0.0000	0.0000	0.0000	0.0000	0.0000	0.0000	0.0000	0.0000	145	A
0.5000	0.5000	0.0000	0.0000	0.0000	0.0000	0.0000	0.0000	0.0000	0.0000	135	A
0.5000	0.5000	0.0000	0.0000	0.0000	0.0000	0.0000	0.0000	0.0000	0.0000	125	A
0.5000	0.5000	0.0000	0.0000	0.0000	0.0000	0.0000	0.0000	0.0000	0.0000	115	A
0.5000	0.5000	0.0000	0.0000	0.0000	0.0000	0.0000	0.0000	0.0000	0.0000	105	A
0.5000	0.5000	0.0000	0.0000	0.0000	0.0000	0.0000	0.0000	0.0000	0.0000	85	M
0.5000	0.5000	0.0000	0.0000	0.0000	0.0000	0.0000	0.0000	0.0000	0.0000	75	M
0.5000	0.5000	0.0000	0.0000	0.0000	0.0000	0.0000	0.0000	0.0000	0.0000	65	M
0.5000	0.5000	0.0000	0.0000	0.0000	0.0000	0.0000	0.0000	0.0000	0.0000	55	M
0.5000	0.5000	0.0000	0.0000	0.0000	0.0000	0.0000	0.0000	0.0000	0.0000	45	M

S3. FEATURE SELECTION

A. Feature construction

1. Features for ferroelectric ceramics

We utilized the same master feature set we have successfully used previously for predicting electrostrains in $BaTiO_3$-based ferroelectric ceramics.[1] Supplementary Table 5 lists the 51 features consisting of properties of the perovskite A-site and B-site elements (atom or ions) including electronegativity, ionic displacement, ideal bond distance. We also defined additional features as ratios (and products) of properties for the A-site and B-site cations according to the following:

$$X_{(A/B)} = X_A/X_B = (f_{Ba}X^{Ba} + f_{Ca}X^{Ca} + f_{Sr}X^{Sr} + f_{Cd}X^{Cd})/(f_{Ti}X^{Ti} + f_{Zr}X^{Zr} + f_{Sn}X^{Sn} + f_{Hf}X^{Hf}) \quad (1)$$

$$X_{(A\cdot B)} = X_A \cdot X_B = (f_{Ba}X^{Ba} + f_{Ca}X^{Ca} + f_{Sr}X^{Sr} + f_{Cd}X^{Cd}) \cdot (f_{Ti}X^{Ti} + f_{Zr}X^{Zr} + f_{Sn}X^{Sn} + f_{Hf}X^{Hf}), \quad (2)$$

where f_{Ba}, f_{Ca}, f_{Sr}, f_{Cd}, f_{Ti}, f_{Zr}, f_{Sn}, f_{Hf} are the mole fractions and X^{Ba}, X^{Ca}, X^{Sr}, X^{Cd}, X^{Ti}, X^{Zr}, X^{Sn}, X^{Hf} correspond to properties associated with each cation, respectively. For simplicity, $X_{(A\cdot B)}$ was abbreviated to lowercase x and $X_{(A/B)}$ was abbreviated to capital X. Taking electronegativity (absolute scale) as an example, "en" refers to $EN_{(A\cdot B)}$ wheras "EN" refers to $EN_{(A/B)}$. Together with the tolerance factor (t) and the phenomenological features NCT and NTO,[1] which define the increase (+1), decrease (-1) or no change (0) of the transition temperatures on doping for the C-T and T-O transitions, we have altogether 95 features for the ferroelectric ceramic system.

2. Features for shape memory alloys

We employed similar features we have previously used for predicting the compositions and transformation temperatures of shape memory alloys.[2] These features are listed in Supplementary Table 6.

Supplementary Table 5 | Material descriptors for $BaTiO_3$ based solid solutions

Material Descriptors	Physical properties
rA	Shannon's ionic radii of A-site
rB	Shannon's ionic radii of B-site
t	Tolerance factor calculated by Shannon's ionic radii
dA-O	Ideal A-O bond distance
dB-O	Ideal B-O bond distance
AEN	A-site electronegativity(absolute scale)
BEN	B-site electronegativity(absolute scale)
AEN-P	A-site electronegativity(Pauling scale)
BEN-P	B-site electronegativity(Pauling scale)
AEN-MB	A-site electronegativity(Matyonov-Batsanov)
BEN-MB	B-site electronegativity(Matyonov-Batsanov)
DA	Ionic displacement of A-site
DB	Ionic displacement of B-site
ACVW	Crystallographic van der Waals radii of A-site element
BCVW	Crystallographic van der Waals radii of B-site element
AEVW	Equilibrium van der Waals radii of A-site element
BEVW	Equilibrium van der Waals radii of B-site element
AD	Density of A-site element
BD	Density of B-site element
AEC	Electrical conductivity of A-site element
BEC	Electrical conductivity of B-site element
ATC	Thermal conductivity of A-site element
BTC	Thermal conductivity of B-site element
AHA	Heat of atomization of A-site element
BHA	Heat of atomization of B-site element
AHF	Heat of fusion of A-site element
BHF	Heat of fusion of B-site element
AEA	Ionization energies of A-site element
BEA	Ionization energies of B-site element
AAV	Atomic volume of A-site element
BAV	Atomic volume of B-site element
AP	Polarizability of A-site element
BP	Polarizability of B-site element
AAR	Atomic radius of A-site element
BAR	Atomic radius of B-site element
AAN	Atomic number of A-site element in element period table
BAN	Atomic number of B-site element in element period table
ARAM	Relative atomic mass of A-site element
BRAM	Relative atomic mass of B-site element
AGR	Group of A-site element in element period table
BGR	Group of B-site element in element period table
APE	Period of A-site element in element period table
BPE	Period of B-site element in element period table
AVEN	A-site valence electron number
BVEN	B-site valence electron number
NCT	(+1,-1,0), the trend(increase, decrease or nochange) of the dependence of T_{C-T} on the doping element
NTO	(+1,-1,0), the trend(increase, decrease or nochange) of the dependence of T_{T-O} on the doping element
ZeffA	Effective nuclear charge of A-site element
ZeffB	Effective nuclear charge of B-site element
TEA	Thermal expansion of A-site element
TEB	Thermal expansion of B-site element

Supplementary Table 6 | Material descriptors for Shape Memory Alloys

Material Descriptors	Physical properties
numa	number of elements
cs	Pettifor chemical scale
arc	Clementi's atomic radii
en	Pauling electronegativity
ven	Valence electron numbers
mr	metallic radius
dor	Waber-Cromer's pseudopotential radii
anum	atomic number
mass	atomic mass
ea	valence electron numbers of average atomic number
volume	volume
Tm	melting point
energy1	First ionisation energy
YM	Young's Modulus
CE	Cohesive Energy (per atom)
EBE	Electron Binding Energy
ccs	average concentration of cs
carc	average concentration of arc
cmr	average concentration of mr
cen	average concentration of en
cven	average concentration of ven
cdor	average concentration of dor
cmass	average concentration of mass
cea	average concentration of valence electron numbers of average atomic number
cvolume	average concentration of volume
cTm	average concentration of volume of element melting point
cenergy1	average concentration of First ionisation energy
cYM	average concentration of Young's Modulus
cCE	average concentration of Cohesive Energy (per atom)

B. Feature selection methods

Including all the features in Supplementary Table 5 and Supplementary Table 6 in our machine learning models increases the dimensionality and computational costs. Moreover, irrelevant, noisy and redundant features can lead to overfitting. Therefore, removing features that are not directly correlated to the target property is essential.

We used three feature selection methods to select relevant features or material descriptors. Gredient Boosting (GB) is used initially to rank the features according to their relative importance to the transition temperature. A further down select is made by eliminating those least correlated as determined by a Pearson map. Finally, we used the method of best subsets (wrap) within the Kriging framework to obtain the final feature sets.

1. Gradient Boosting (GB)

The large number of features are not all equally relevant and only a few with substantial influence on the target are good to be used to set up the model. Assessing the contribution of each feature is conducive to removing redundant ones. Gradient Boosting (GB) is a good choice for this purpose. The squared relevance for each feature x_l calculated as follows:

$$\mathcal{I}_l^2(T) = \sum_{t=1}^{J-1} \hat{\boldsymbol{\iota}}_t^2 \boldsymbol{I}(\boldsymbol{\nu}(t) = l), \tag{3}$$

where $J-1$ is the internal nodes of the tree, $\boldsymbol{\iota}_t^2$ is maximal estimated improvement in squared error risk. The squared relative importance of feature x_l is the sum of such squared improvements over all internal nodes chosen as the splitting variable. The parameter ν, the shrinkage rate, can control the learning rate of the boosting procedure. Due to the stabilizing effects of averaging in additive tree expansions, we use the following equation to substitute for its counterpart for a single tree.

$$\mathcal{I}_l^2 = \frac{1}{M} \sum_{m=1}^{M} \mathcal{I}_l^2(T_m), \tag{4}$$

where M is the number of boosting iterations. A large M causes an arbitrarily small training risk but may lead to "overfitting". Small values of ν result in larger training risk but favor better test error.[3] Therefore, a trade-off between the two is preferred for our case.

2. Pearson Map

We keep several features according to their importance evaluated by GB. The Pearson product-moment correlation coefficient (PMCC) measures the degree of correlation amongst the features using the following equation,

$$r = \frac{\sum_{i=1}^{n}(x_i - \overline{x}) \sum_{i=1}^{n}(y_i - \overline{y})}{\sqrt{\sum_{i=1}^{n}(x_i - \overline{x})^2 (y_i - \overline{y})^2}}, \tag{5}$$

where x_i and y_i are the values of two features corresponding to the i th composition; $\bar{x} = \sum_{i=1}^{n}(x_i)$ and $\bar{y} = \sum_{i=1}^{n}(y_i)$ are the mean of x_i and y_i; n is the sum of the number of samples in the training dataset. The PMCC r is between [-1 , +1]. The absolute value of r approaching 1 connotes a high correlation between variables.

3. Best Subset

After the selection by GB (*i.e.*, an embedded method) and Pearson correlation (*i.e.*, a filter), the number of selected features are still large for modeling. We therefore employed Best subset regression in the Kriging framework to find the subset (the size of the subset $k \in \{1, 2, ..., m\}$) that gives the smallest Leave One Out Cross Validation(LOOCV) Error. The formula for CVerror is given by:

$$CVerror = \sqrt{\frac{1}{n} \sum_{i=1}^{n} (y_i - \hat{y_i})^2}, \tag{6}$$

where n is the number of observations in the training dataset, y_i is the measured value and $\hat{y}_i$ is the predicted value based on n-1 observations (leave one out). By repeating this method n times, the average of the squared errors gives CVerror.

C. Feature selection in ferroelectric ceramics

For *Approach 2*, the 95 features for ceramics were ranked by GB and the top 10 (or 15) features were selected for the Pearson filter, as shown in the top panels of Supplementary Figure 2. For the τ(Para-Ferro) tranisition, 10 features including NCT, BAV, BD, BAN, EN, BRAM, TA.B, BEA, av, te were chosen (TA.B is the ratio of Shannon's ionic radii of the A-site cation and B-site cation). For τ(T-O), 15 features including z, NTO, d1, D, pe, tA.B, enmb, p, an, TC, rB, av, ar, EC, HF were chosen. For τ(T-O), 15 features including tA.B, rB, enmb, BAV, BEVW, DB, BD, BCVW, AHA, z, ar, BPE, av, an, NCT were chosen.

The Pearson correlation coefficient r for the above sets of features was calculated and is presented as a color gradient heat-map shown in the middle panels of Supplementary Figure 2. The Red/blue indicate a negative/positive correlation between two features. The deeper the color or the higher the saturation of the pie, the stronger the correlation between features. The features with Pearson correlation coefficient $r \geq 0.9$ are considered highly correlated and only one of them is retained. For example, BAN, BRAM and BD are highly correlated and BD is chosen as the feature left. The features BD, NCT, BAV, BD, EN, TA.B, BEA, av, te are chosen for τ(Para-Ferro), the features of z, enmb, NTO, pe, tA.B, rB, HF for τ(T-O) and the features tA.B, rB, enmb, BAV, BEVW, z, av, an, NCT for τ(O-R).

The bottom panels of Supplementary Figure 2 show the CVerror values of all the subset models for the three phase transition boundaries: τ(Para-Ferro), τ(T-O) and τ(O-R), respectively. The red frontier tracks the best model for a given number of features. The error decreases with increasing number of features. However, there is little improvement in CVerror beyond the use of 4 features. Moreover, overfitting is a possibility if we include more features. Hence, we limit ourselves to not more than 4 features in the subsequent learning process. The final sets of features for τ(Para-Ferro), τ(T-O) and τ(O-R) are (BD, EN, TA.B, av), (NTO, rB, HF) and (z, av, rB, enmb), respectively. If the number of features is limited to 3, the final sets of features for τ(Para-Ferro), τ(T-O) and τ(O-R) include (EN, TA.B, av), (NTO, rB, HF) and (BEVW, an, NCT), respectively.

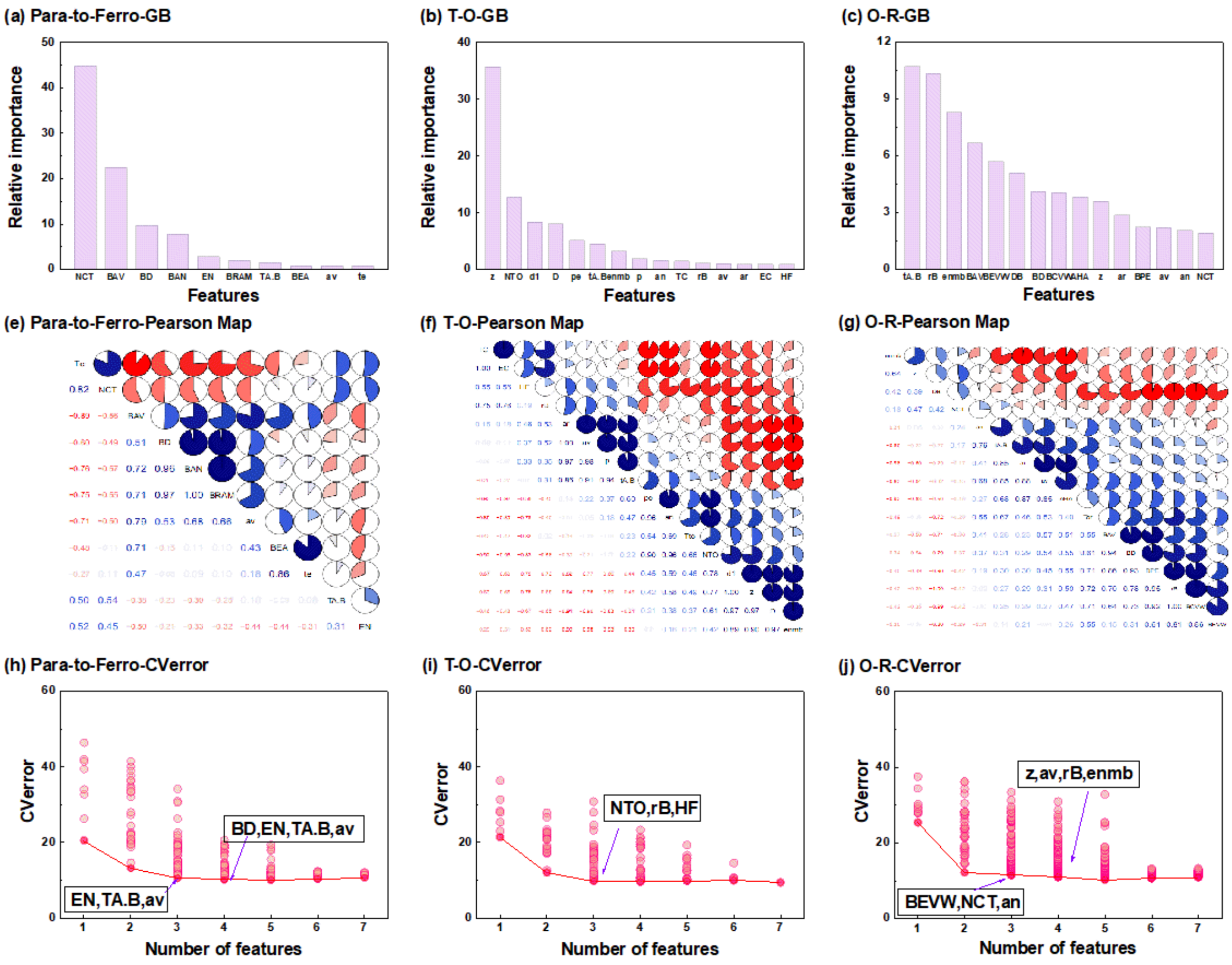

Supplementary Figure 2 | Feature selection for three regressors for the transitions τ(Para-Ferro), τ(T-O) and τ(O-R) in $BaTiO_3$ based ceramics. The top row is the result of Gradient Boosting, which ranks the features according to their relative importance to the properties. Only the first 10 or 15 features are shown and selected. The middle row is the Pearson correlation map (pie chart), which further filters the highly correlated features (correlation coefficient ≥ 0.9). The bottom row are the results of CVerror as a function of numbers of features for all the subsets of the features retained from the Pearson map. The red frontier tracks the model with the smallest CVerror for a given number of features. The arrow points to the features that can be selected based on a trade-off between minimizing CVerror as well as the numbers of features.

D. Feature selection in shape memory alloys

As shown in Supplementary Figure 3, the features for τ(A-M) are down-selected following the same procedure as for $BaTiO_3$ based ceramics. The selected set of features include anum, energy1, YM. Due to the relatively small training data for τ(M1-M2) (15 samples), only the Pearson Map method is employed to filter the features for τ(M1-M2). The Pearson Map is shown in Supplementary Figure 4. After removing the redundant features, 4 features including numa, cs, cven and EBE were used. The down-selected features or material descriptors for the phase boundaries in the

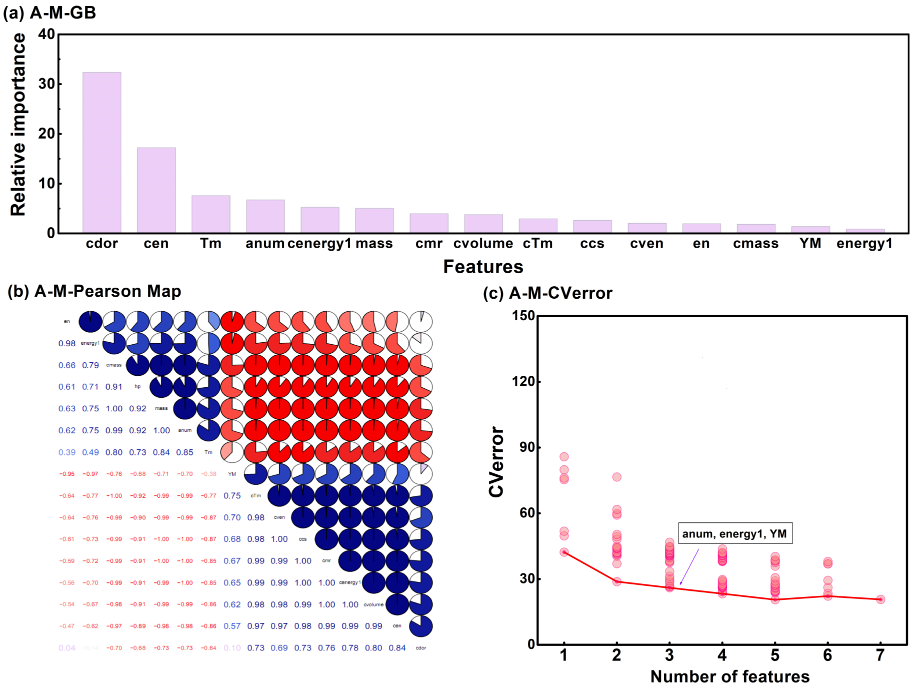

Supplementary Figure 3 | Feature selection for use in regression for τ(A-M) in NiTi base shape memory alloys. (a) the results of Gradient Boosting, which ranks the features according to their relative importance to the properties are shown. Only the top 15 features are shown and selected. (b) the Pearson correlation map (pie chart), which further filters the highly correlated features (correlation coefficient ≥ 0.95). (c) the results for CVerror as a function of numbers of features for all the feature subsets from the Pearson map. The red frontier tracks the model with the smallest CVerror for a given number of features. The arrow points to the feature set chosen to balance the trade-off between CVerror and numbers of features.

phase diagrams of $BaTiO_3$ based ceramics and NiTi based SMAs (*Approach2*) are shown in Supplementary Table 7.

The final features used in *Approach 1a* and *Approach 1b* were chosen in much the same way as for *Approach2*, that is, by employing Gradient Boosting and the Pearson map. The selected material descriptors for phase prediction using classification (*Approach 1a*) are shown in Supplementary Table 8. The selected material descriptors for transition temperature prediction using classification data (*Approach 1b*) are shown in Supplementary Table 9.

Supplementary Table 7 | The selected material descriptors for phase transitions (*Approach 2*) in $BaTiO_3$ based solid solutions and SMAs

Phase boundary	Down-selected material descriptors for each transition	Down selected descriptors if three selected
τ(Para-Ferro)	BD, EN, TA.B, av	EN,TA.B,av
τ(T-O)	NTO, rB, HF	
τ(O-R)	z, av, rB, enmb	BEVW,NCT,an
τ(A-M)	anum, energy1, YM	
τ(M1-M2)	numa,cs,cven,EBE	

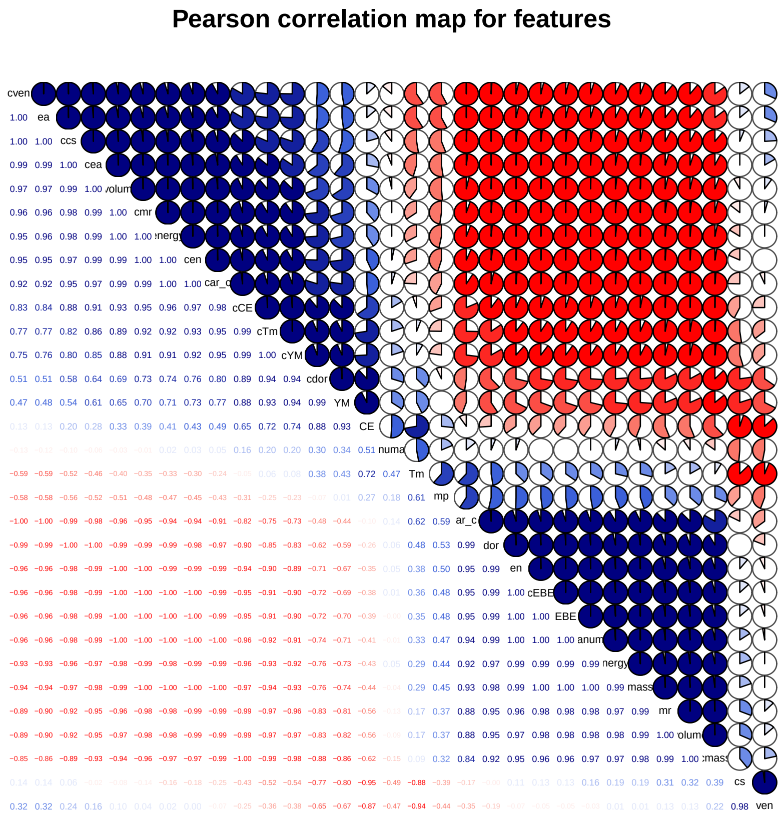

Supplementary Figure 4 | Pearson correlation map of features for τ(M1-M2), a graphical representation of the Pearson correlation matrix for the initial features. Blue and red colors indicate positive and negative correlations, respectively. The lighter the tone, the less significant is the corresponding correlation. The filled fraction of the circles in each of the pie charts correspond to the absolute value of the associated Pearson correlations coefficient. (For interpretation of the references to the color in this figure legend, the reader is referred to the web version of this article.)

Supplementary Table 8 | The selected material descriptors for phase prediction using classification (*Approach 1a*)

System	Down-selected material descriptors for each system
$BaTiO_3$ based solid solutions	T, BD, BAV, NCT, EN
SMAs	T, cenergy1, cdor, CE

Supplementary Table 9 | The selected material descriptors for transition temperature prediction using classification data (*Approach 1b*)

Phase boundary	Down-selected material descriptors for each transition
τ(Para-Ferro)	NCT, BAV, av, BAN
τ(T-O)	BAV, z, av, pe
τ(O-R)	BCVW, tA.B, BAV, DB
τ(A-M)	ea, cdor, YM, CE
τ(M1-M2)	cdor, ea, CE, cs

S4. CLASSIFICATION IN (*APPROACH 1A*)

A. Phase diagram predicted by classification (*Approach 1a*)

We employ both Random Forest classifier(RF) and Support Vector Machine(SVM) for comparison and study their robustness to data requirements. Here we take the $Ba(Ti_{1-\omega}Zr_{\omega})O_3$ as an example to show how these two classifiers perform in predicting the presence of phase boundaries. Supplementary Figure 5 shows how we obtain classification training data by using the $Ba(Ti_{1-\omega}Zr_{\omega})O_3$ transition temperature data in the initial database. The temperature is discretized into integer values for each composition with a step size of 10^oC and a crystal structure is labeled at a given temperature within the temperature range between transition temperatures. The data for $BaTiO_3(\omega = 0)$ are shown in Supplementary Table 2 with a virtual space created by imposing a mesh in the temperature-composition plane. The phases of compounds for a given temperature, discretized by a step size of 10^oC, and composition are then predicted using both classifiers, and the results are shown in Supplementary Figure 6.

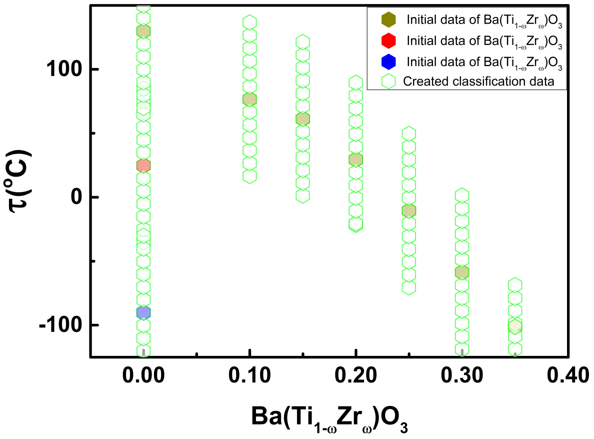

Supplementary Figure 5 | Classification training data for the $Ba(Ti_{1-\omega}Zr_{\omega})O_3$ phase diagram. The solid symbols are data on the phase boundary and the different colors stand for different phase transitions. The temperature is discretized into integer values for each composition with the step size 10^oC, and a particular crystal structure is labeled at a given temperature within a temperature range between transition temperatures.

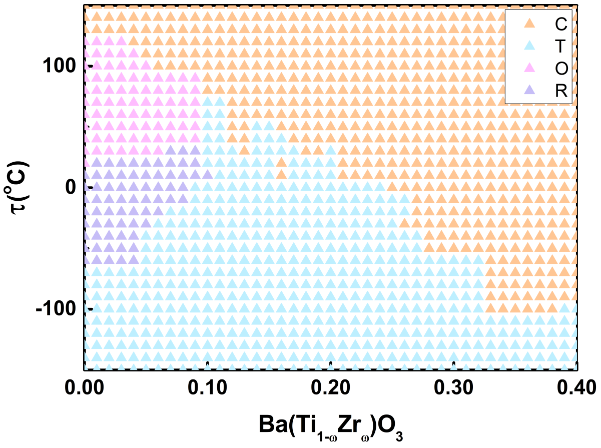

Supplementary Figure 6 | $Ba(Ti_{1-\omega}Zr_{\omega})O_3$ phase diagram established by a Random Forest classifier. The discretized points in the phase diagram indicate the phase diagram virtual space and the different colors refer to the different phases.

The predictions for the $Ba(Ti_{1-\omega}Zr_{\omega})O_3$ phase diagram using Support Vector Machine are shown in Supplementary Figure 7(a-c) and those with Random Forest in Supplementary Figure 7(e-g). The smoothness of the phase boundaries is determined by the discretization and number of training points used. Compared with Supplementary Figure 6, the

temperature is discretized by a smaller step size of 1°C in Supplementary Figure 7. In *Approach 1*, we generate a data set of 3968 points for ceramics. We use 100% of the data, 50% and 20% to predict the phase diagram corresponding to (a,e), (b,f) and (c,g) of Supplementary Figure 7, respectively. As the number of training data points decreases, the accuracy suffers but we find that a data size of 50% (1984 points) with SVM essentially captures this direct classification strategy.

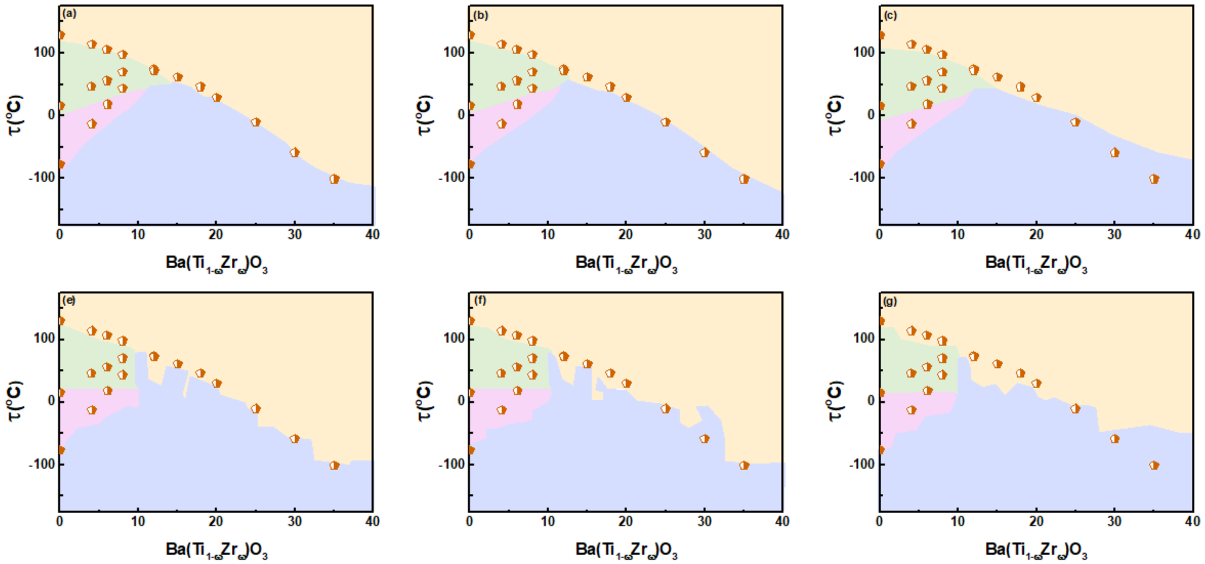

Supplementary Figure 7 | Ba(Ti$_{1-\omega}$Zr$_\omega$)O$_3$ phase diagram established by Support Vector Machine in Supplementary Figure 7(a-c) and Random Forest classifier in Supplementary Figure 7(e-g). The coded colors in the phase diagram show the different phases. Panels (a,e) with 100% (3968 points), (b,f) with 50% and (c,g) with 20% of training data used. A data size of 1984 (50%) using SVM essentially captures this direct classification strategy.

B. Model Performance for *Approach 1a*

Approach 1a predicts directly the phase at any given temperature via a classifier using in total 3986 data points from our ceramic database and 2720 data points from the SMAs database. The uncertainties from the SVM classifier were calculated using 10-fold cross validation and were 0.870 for ceramics and 0.886 for SMAs. The data was split into 90% for training and 10% for testing. The resulting receiver operating characteristic (ROC) curves are shown in Supplementary Figure 8, for each phase using a one-vs-all strategy indicate the accuracy of the classifier. The area under the ROC curve is equal to the probability that a classifier will rank a randomly chosen positive instance higher than a randomly chosen negative one (assuming 'positive' ranks higher than 'negative').[4] The areas under the ROC curves for the different phases in the two systems were: 0.988 for B2, 0.995 for B19, 0.976 for B19', 0.994 for C, 0.977 for T, 0.953 for O and 0.967 for R. The large area above 0.95 ensures an accurate and robust classification.

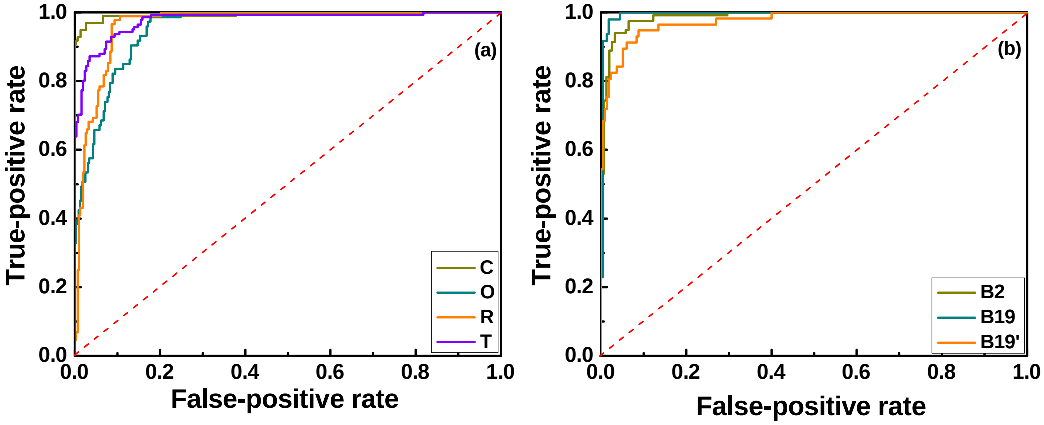

Supplementary Figure 8 | The receiver operating characteristic (ROC) curves in (a) and (b) indicate excellent area coverage suggesting good performance in distinguishing different phases for our SVM classifier for the $BaTiO_3$ base solid solution and SMA system.

S5. MODEL PERFORMANCE

A. Model Performance for *Approach 2*

In *Approach 2* we employ Universal Kriging to interpolate values and regress phase boundaries from relatively few data points. Kriging is a spatial interpolation method providing robust estimation of targets and uncertainties associated with unknown states. It provides a foundation for estimating phase boundaries and the subsequent optimization of phase diagrams.

Because we are using experimental observations, we consider noisy measurements in the Kriging models as follows. The observation $\tilde{y}$ has a noise contribution, *i.e.*, $\tilde{y} = y(x) + \epsilon$, where ϵ is a realization of a random variable ε_j. We here assume that the noise obeys a Gaussian distribution, $\varepsilon \sim \mathcal{N}(0, \tau^2)$, and set τ^2 to be 10. y is a realization of a Gaussian process Y following Universal Kriging.

$$Y = \mathbf{m} + Z = \sum \beta \mathrm{f} + Z, \tag{7}$$

where $\mathbf{m}$ is a trend function, β is the coefficient and the process Z is assumed Gaussian.

Assume there are p pieces of training data x^* and unknown data points shown as x. The Universal Kriging(UK) equations are given by:[5,6]

$$\mu = \mathbf{m}(x) + K(x, x^*)(K + \mathbf{\Delta})^{-\mathbf{1}}(\tilde{y} - \mathbf{m}(x^*)), \tag{8}$$

$$\begin{aligned} s^2 = s^2_{SK} + (\mathrm{f}(x)^\intercal - K(x,x^*)^\intercal (K+\Delta)^{-1}\mathrm{f}(x^*))^\intercal (\mathrm{f}(x^*)^\intercal \\ (K+\Delta)^{-1}\mathrm{f}(x^*))^{-1}(\mathrm{f}(x)^\intercal - K(x,x^*)^\intercal (K+\Delta)^{-1}\mathrm{f}(x^*)), \end{aligned} \tag{9}$$

where $\tilde{y}$=$(\tilde{y}_1, ..., \tilde{y}_p)^T$, K is covariance between train data points, $\mathbf{\Delta}$ is the diagonal matrix with the diagonal terms as $\tau_1^2, ..., \tau_p^2$. And s^2_{SK} is variance given by Simple Kriging(SK), the formula is shown by:

$$s^2_{SK} = K(x,x) - K(x,x^*)(K + \mathbf{\Delta})^{-\mathbf{1}}K(x^*, x), \tag{10}$$

The kernel with $g(h) = exp(-\frac{1}{2}(\frac{h}{\theta})^2)$(Gaussian kernel) is set as the covariance kernel to obtain the covariance of the data. Here h and θ the hyper-parameters of the Kriging model, which are both *characteristic length-scales*. We thus can estimate the mean value of the phase transition temperature and associated uncertainties.

We compare the predicted values with the measured values to track the performance of the Universal Kriging model on our datasets. The predicted transition temperature values of all the compounds in the database are determined by the leave one out (LOO) method. Each time we use $n-1$ observations to train and predict the left out sample. We repeat this procedure n times to obtain the predicted values for all observations. The predicted transition temperature is plotted as a function of measured in Supplementary Figure 9 and Supplementary Figure 10 for ceramics and alloys, respectively. For a perfect model, the predicted values should be exactly the same as the measured values and all the data points will fall along the 45° diagonal line. Supplementary Figure 9 suggests that models for ceramics perform reasonably well with calculated Leave One Out CVerrors for τ(Para-Ferro), τ(T-O) and τ(O-R) of 10.17, 9.83, and 11.73^oC, respectively. The performances of SMAs models shown in Supplementary Figure 10 are not as good as those for ceramics. The principal reason is that we have a relatively small amount of training data containing experimental noise. The Leave One Out CVerrors for τ(A-M) and τ(M1-M2) were 26 and 57.57^oC, respectively. We define the ratio of RMSE and y range by the formula:

$$Ratio_{LOOCV} = \frac{RMSE}{max_{measured} - min_{measured}}, \tag{11}$$

Thus, the $Ratio_{LOOCV}$ for τ(Para-Ferro), τ(T-O) and τ(O-R) is 4.4%, 5.92%, and 8.04%, respectively for ceramics and τ(A-M) and τ(M1-M2) are 5.67% and 20.60% respectively for SMAs.

B. Model Performance for *Approach 1b*

Support Vector Regression was employed to predict the phase boundaries in *Approach 1b*. What is different from *Approach 2* is that the input are the support vectors that result from the classification model, in contrast to using the transition temperature data. The training data are divided into two parts or classes to obtain an estimate of the

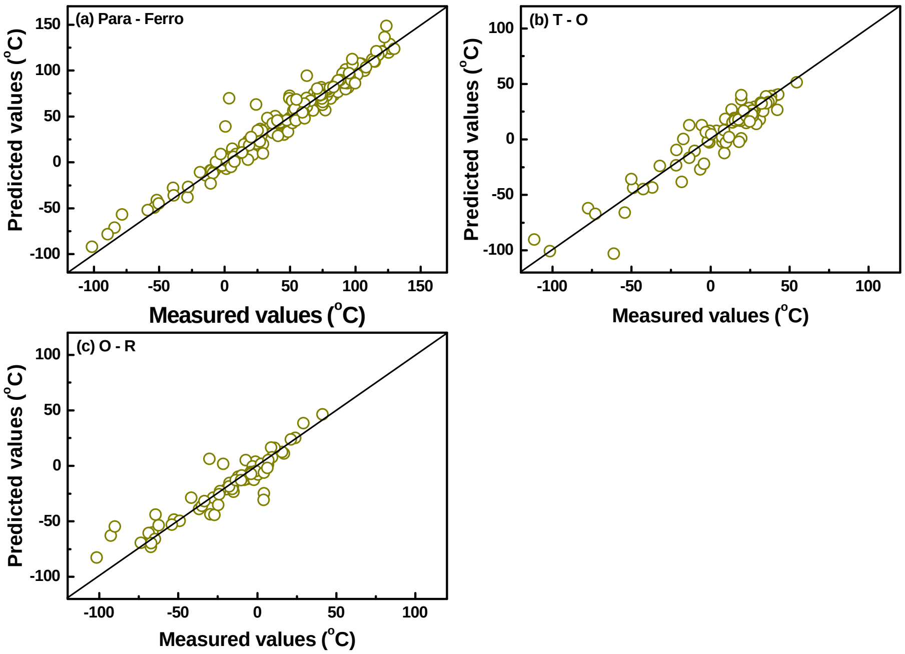

Supplementary Figure 9 | Performance of the Universal Kriging model to predict the three phase transitions in $BaTiO_3$ based solid solutions using Leave One Out.

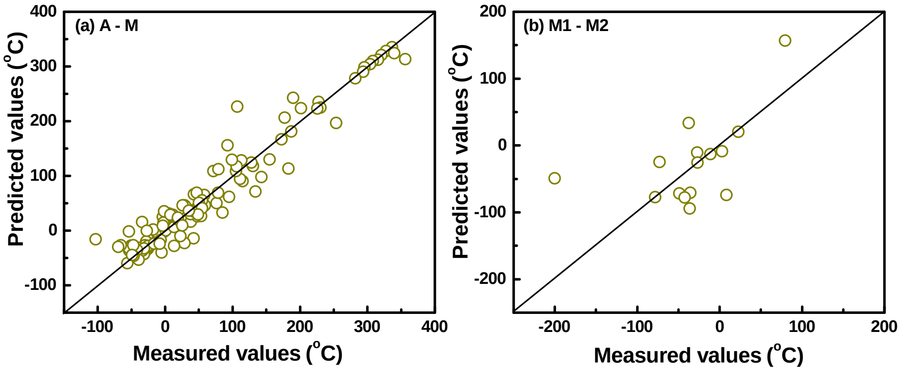

Supplementary Figure 10 | Performance of the Universal Kriging model to predict the two phase transitions in shape memory alloys using Leave One Out (LOO). The relatively small data set available for the M1-M2 transition is reflected in (b).

margin using binary classification. The classes are C *and* T+O+R for the Para-to-Ferro phase boundary capturing the C-T and C-R transitions, C+T *and* O+R for the T-O phase boundary, and C+T+O *and* R for the O-R phase boundary. A support vector machine attempts to find the line that "best" separates two classes of points. That is, the line that results in the largest margin between the two classes and the points that lie on this margin are the support vectors. They provide an estimate of the hyperplane that best separates the two classes and for us this would be an estimate for the transition temperatures from one phase to another. For the Para-to-Ferro transition, the two classes C *and* T+O+R have 1086 and 2900 data points, respectively. The data set is thus reasonably balanced and leads to 748 support vectors. By interpreting the support vectors as predictions of transition temperatures, we can next regress this data to predict a full phase boundary, which are the solid lines shown in Supplementary Figure 3b for all the transitions. We use support vector regression (SVR) with 748, 891, 990 support vectors giving a leave one out (LOO) cross validation (CV) error of 5.54% for the Para-to-Ferro transition, 7.10% for the T-O and 5.09% for O-R transition in the ceramic system, respectively. 749 and 562 support vectors are used in SVR model and $Ratio_{LOOCV}$ by 5.32% and 7.47% for τ(A-M) and τ(M1-M2) for the SMA system are calculated.

S6. PHASE DIAGRAM FOR THE CERAMIC SYSTEM: (1-ω)BATIO$_3$-ωBA(TI$_{0.6}$ZR$_{0.22}$SN$_{0.01}$HF$_{0.17}$)O$_3$

We search all the phase diagrams for the system (1-ω)BaTiO$_3$-ωBa(Ti$_{1-x-y-z}$Zr$_x$Sn$_y$Hf$_z$)O$_3$, which includes a search space with 11680 phase diagrams. The aim is to find a compound undergoing a C to R transition with a transition temperature as as high as possible. We find that (1-ω)BaTiO$_3$-ωBa(Ti$_{0.6}$Zr$_{0.22}$Sn$_{0.01}$Hf$_{0.17}$)O$_3$ has multi-transitions for compositions $\omega < 0.27$, and only a C-R transition for compositions $\omega > 0.27$. This is obtained by first scanning the phase diagram virtual space to calculate the temperature where T_B of the para-to-ferro phase boundary intersects with the O-R phase boundary for each phase diagram, where T_B is the set of multi-phase coexistence temperatures. We rank T_B from large to small and choose the phase diagram with the largest value T_B^{max}. We find about 200 phase diagrams with multi-phase coexistence point temperatures which do not differ much from T_B^{max}, and in principle any of them could be selected as our target phase diagram Here we consider one of them with the predicted phase diagram shown in Supplementary Figure 11(a).

Our machine learning predictions so far inevitably contain uncertainties and therefore the validation and optimization of the predicted phase diagrams through experiments is a crucial element of our approach. For phase diagram, values of ω correspond to different compositions that can be experimentally synthesized and characterized. The question is how do we choose the optimal compositions so that the number of new experiments that need to be carried out can be minimized. In this work we selected the next experiment by maximizing a utility function, ($\mathcal{U}_\omega$), which tracks the variance in the predictions from the machine learning for each possible experiment. That is, we choose "Maximum Variance" according to $arg_{max}(\mathcal{U}_\omega) = arg_{max}(\mathcal{V}_\omega)$, where $\mathcal{V}_\omega$ is the variance of the predictions for composition ω.

The predicted values are consistent with the measured ones as shown in Supplementary Figure 11(e) and the overall uncertainties decrease with iterations (number of experiments) as shown in Supplementary Figure 11(f).

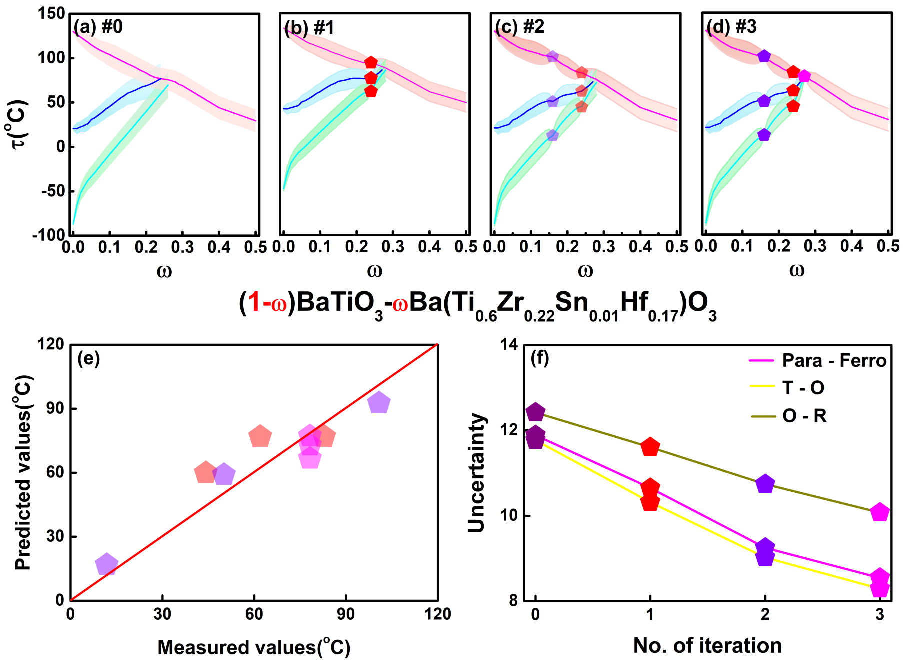

Supplementary Figure 11 | The rapid validation and optimization of preselected phase diagram of (a) (1-ω)BaTiO$_3$-ωBa(Ti$_{0.6}$Zr$_{0.22}$Sn$_{0.01}$Hf$_{0.17}$)O$_3$. The panels (b) - (d) are the phase diagrams after subsequent iterations. In each iteration, a particular composition is chosen by a utility function, that is, "Maximum Variance", and the compound is synthesized and characterized. The results augmented the dataset and a new iteration comences. (e) shows that the predictions of phase boundaries are consistent with the measured values after each iteration as all points approximately fall along the diagonal line. (f) shows that the overall uncertainty associated with the phase boundary decreases with iterations.

S7. EXPERIMENTAL MEASUREMENTS

We decide on the next candidate to measure based on an active learning strategy using "Maximum Variance". Supplementary Table 10 lists the compositions recommended for each iteration. The compounds are synthesized and characterized to obtain the phase transition data, which feeds back into the database. The Martensite-Austenite phase transition temperatures are measured by Differential scanning calorimetry(DSC). The results are shown in Supplementary Figure 12. The temperatures of the peak on heating are selected as the phase transition temperatures. The phase transitions in $BaTiO_3$ base solid solutions are determined by the temperature dependence of the dielectric permittivity. Supplementary Figure 13 (a) and (b) show the results for (1-ω)$BaTiO_3$-$\omega$$Ba(Ti_{0.6}Zr_{0.22}Sn_{0.01}Hf_{0.17})O_3$ and (1-ω)$Ba_{0.61}Ca_{0.28}Sr_{0.11}TiO_3$-$\omega$$BaTi_{0.888}Zr_{0.0616}Sn_{0.0028}Hf_{0.0476}$, respectively.

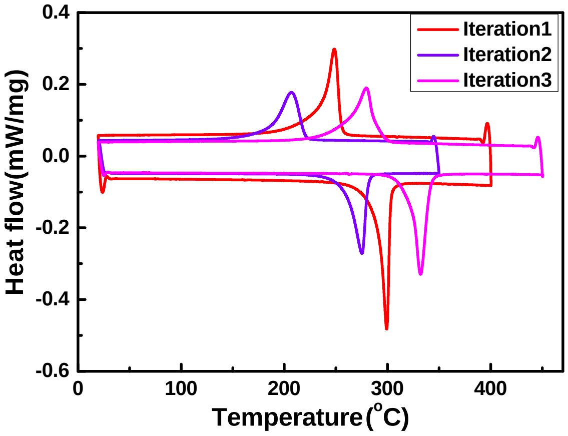

Supplementary Figure 12 | DSC curves of compositions $Ti_{0.309}Ni_{0.485}Hf_{0.161}Zr_{0.0246}Nb_{0.0204}$ (Iteration #1), $Ti_{0.309}Ni_{0.485}Hf_{0.135}Zr_{0.037}Nb_{0.034}$ (Iteration #2), $Ti_{0.309}Ni_{0.485}Hf_{0.1818}Zr_{0.01468}Nb_{0.00952}$ (Iteration #3).

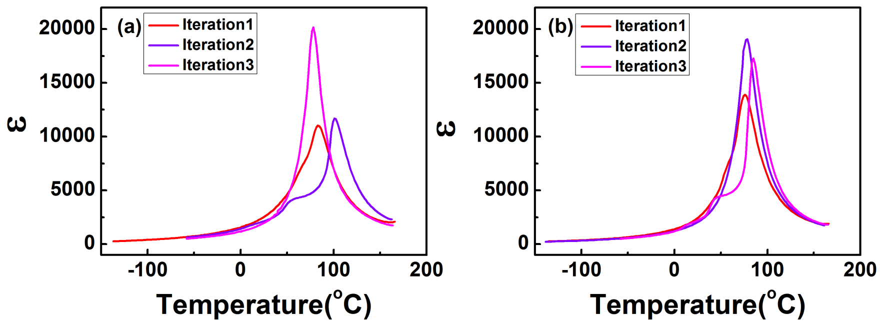

Supplementary Figure 13 | Temperature dependence of dielectric permittivity for the ceramics (measured at 10 kHz). (a) and (b) are results for (1-ω)$BaTiO_3$-$\omega$$Ba(Ti_{0.6}Zr_{0.22}Sn_{0.01}Hf_{0.17})O_3$ and (1-ω)$Ba_{0.61}Ca_{0.28}Sr_{0.11}TiO_3$-$\omega$$BaTi_{0.888}Zr_{0.0616}Sn_{0.0028}Hf_{0.0476}$.

Supplementary Table 10 | The recommended composition by active learning in subsequent iterations.

Iterations	compositions in SMAs phase diagram	compositions in B-site $BaTiO_3$ base phase diagram	compositions in AB site $BaTiO_3$ base phase diagram
1	$Ti_{0.309}Ni_{0.485}Hf_{0.161}Zr_{0.0246}Nb_{0.0204}$	$Ba(Ti_{0.904}Zr_{0.0528}Sn_{0.0024}Hf_{0.0408})O_3$	$(Ba_{0.9571}Ca_{0.0308}Sr_{0.0121})(Ti_{0.90032}Zr_{0.054824}Sn_{0.002492}Hf_{0.042364})O_3$
2	$Ti_{0.309}Ni_{0.485}Hf_{0.135}Zr_{0.037}Nb_{0.034}$	$Ba(Ti_{0.936}Zr_{0.0352}Sn_{0.0016}Hf_{0.0272})O_3$	$(Ba_{0.9883}Ca_{0.0084}Sr_{0.0033})(Ti_{0.89136}Zr_{0.059752}Sn_{0.002716}Hf_{0.046172})O_3$
3	$Ti_{0.309}Ni_{0.485}Hf_{0.1818}Zr_{0.01468}Nb_{0.00952}$	$Ba(Ti_{0.892}Zr_{0.0594}Sn_{0.0027}Hf_{0.0459})O_3$	$(Ba_{0.9142}Ca_{0.0616}Sr_{0.0242})(Ti_{0.91264}Zr_{0.048048}Sn_{0.002184}Hf_{0.037128})O_3$

References

* xuedezhen@xjtu.edu.cn
† zhouyumei@xjtu.edu.cn
‡ turablookman@gmail.com

[1] R. Yuan, Z. Liu, P. V. Balachandran, D. Xue, Y. Zhou, X. Ding, J. Sun, D. Xue, and T. Lookman, ADVANCED MATERIALS **30** (2018), 10.1002/adma.201702884.
[2] D. Xue, D. Xue, R. Yuan, Y. Zhou, P. V. Balachandran, X. Ding, J. Sun, and T. Lookman, Acta Materialia **125**, 532 (2017).
[3] T. Hastie, R. Tibshirani, and J. Friedman, "Boosting and additive trees," in *The Elements of Statistical Learning: Data Mining, Inference, and Prediction* (Springer New York, New York, NY, 2009) pp. 337–387.
[4] T. Fawcett, Pattern recognition letters **27**, 861 (2006).
[5] C. E. Rasmussen and C. K. I. Williams, *Gaussian Processes for Machine Learning (Adaptive Computation and Machine Learning)* (MIT Press, 2005).
[6] O. Roustant, D. Ginsbourger, and Y. Deville, Journal of Statistical Software **51** (2013), 10.18637/jss.v051.i01.
